# Plasminogen Activator Inhibitor 1 Controls Abdominal Aortic Aneurism Formation via the Modulation of TGF‐β/Smad2/3 Signaling in Mice

**DOI:** 10.1096/fj.202403133RR

**Published:** 2025-05-05

**Authors:** Mantong Zhao, Lina Hu, Zhuo Lin, Xueling Yue, Xintong Zheng, Meiling Piao, Xianglan Jin, Limei Piao, Rihua Cui, Meilan Liu, Xian Wu Cheng

**Affiliations:** ^1^ Department of Cardiology and Hypertension, Jilin Provincial Key Laboratory of Stress and Cardiovascular Disease Yanbian University Hospital Yanji Jilin P.R. China; ^2^ Department of Public Health Guilin Medical College Guilin Guangxi P.R. China; ^3^ Department of Anesthesiology Yanbian University Hospital Yanji Jilin P.R. China; ^4^ Department of Community Healthcare & Geriatrics Nagoya University Graduate School of Medicine Nagoya Aichike Japan

**Keywords:** abdominal aortic aneurysm, apoptosis, epigallocatechin‐3‐gallate, inflammation, oxidative stress, plasminogen activator inhibitor 1

## Abstract

Given that plasminogen activator inhibitor 1 (PAI‐1) plays an important role in human pathobiology and epigallocatechin‐3‐gallate (EGCG) exerts vasculoprotective actions, we investigated the role(s) of PAI‐1 and the protective effect of EGCG in the mechanism of AAA formation, with a focus on inflammation, oxidative stress, proteolysis, and apoptosis in vivo and in vitro. Nine‐week‐old wild‐type mice (PAI‐1^+/+^) and PAI‐1 deficiency mice (PAI‐1^−/−^) randomly assigned to the sham operation (0.9% saline) and AAA induction (calcium chloride) and subjected to biological and morphological analysis after four weeks. On operative day 28, the AAA lesions had decreased levels of PAI‐1 mRNA and protein. As compared with AAA‐PAI‐1^+/+^ mice, PAI‐1 deficiency aggravated AAA formation accompanied by plasma TNF‐α and IL‐1β elevations. PAI‐1^−/−^ resulted in harmful changes in the levels of gp91^phox^, cleaved‐caspase 8, TGF‐β, p‐Smad2/3, collagen I/III, gp91^phox^, ICAM‐1, VCAM‐1 mRNAs and/or protein in the AAA lesions as well as oxidative stress production and macrophage infiltration. PAI‐1^−/−^ also increased elastin degradation and collagen accumulation associated with the reduction of proteolytic MMP‐2/‐9 expressions and activities. While EGCG reversed the above changes and upregulated PAI‐1 expression. In vitro, PAI‐1 inhibition (silencing and pharmacological inhibitor) and overexpression, respectively, increased and lowered oxidative stress‐induced VSMCs apoptosis and investigated extracellular protein turnover‐related protein changes. These results suggested that the protective role of PAI‐1 and EGCG in AAA formation is based on their ability to inhibit inflammation, oxidative stress, and apoptosis. Moreover, EGCG‐mediated PAI‐1 induction might provide a potential pharmacological treatment for AAA

## Graphical abstract

Graphical illustration of the mechanism underlying the acceleration of plasminogen activator inhibitor1 deletion‐mediated abdominal aneurysm formation in mice undergoing calcium chloride2 induction surgery. Genetic intervention targeted toward plasminogen activator inhibitor1 accelerated AAA formation via the modulation of TGF‐β/Smad2/3 signaling in mice treated with calcium chloride2.

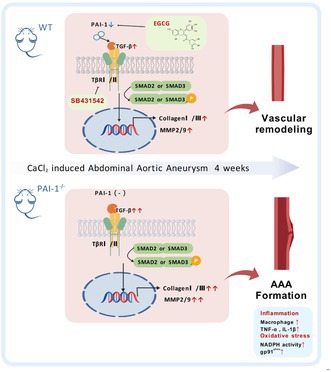

**Abbreviations**
AAA: abdominal aortic aneurysm
BCA: bicinchoninic acid
BSA: bovine serum albumin
BW: body weight
C‐casp8: cleaved‐caspase 8
DMEM: Dulbecco's modified Eagle's medium
ECM: extracellular matrix
ECs: endothelial cells
EGCG: epigallocatechin‐3‐gallate
ELISA: enzyme‐linked immunosorbent assay
EVG: Elastic Van Gieson
FBS: fetal bovine serum
G418: Geneticin 418
H&E: hematoxylin and eosin
ICAM‐1: intercellular adhesion molecule‐1
IL‐1β: interleukin‐1β
Lipo3000: Lipofectamine 3000
MAVSMCs: mouse aortic vascular smooth muscle cells
MMP‐2: matrix metalloproteinase‐2
MMP‐9: matrix metalloproteinase‐9
NADPH: nicotinamide adenine dinucleotide phosphate
NS: normal saline (0.9% NaCl)
PAI‐1: plasminogen activator inhibitor 1
SDS: sodium dodecyl sulfate
SERPINE: serine protease inhibitor
TGF‐β: transforming growth factor‐β
TNF‐α: tumor necrosis factor‐α
tPA: tissue plasminogen activators
TUNEL: TdT‐mediated dUTP nick‐end labeling
uPA: urokinase plasminogen activators
VCAM‐1: vascular cell adhesion molecule‐1
VSMCs: vascular smooth muscle cells
WT: wild‐type

## 1 Introduction

Abdominal aortic aneurysm (AAA) is an aneurysm that forms in the abdominal aorta, a local expansion of the abdominal aortic wall with more than 1.5 times the normal diameter. The presence of AAA poses a significant threat to human health due to its characteristics of irreversible rupture [1]. AAA occurs mainly in older men, typically over 65 [2]. The main risk factors for AAA include sex, smoking, family history, hypertension, and hyperlipidemia. Quitting smoking and lowering blood pressure and cholesterol can help to decrease the occurrence of AAA [2, 3, 4]. The pathogenesis of AAA primarily involves chronic inflammation of the vascular wall, abnormal proliferation and apoptosis of smooth muscle cells, upregulation of matrix metalloproteinases, degradation of extracellular matrix, and oxidative stress [5, 6, 7, 8]. The current understanding of AAA's specific mechanism remains limited, which needs further exploration.

Plasminogen activator inhibitor 1 (PAI‐1), a secret protein, is a member of the serine proteinase inhibitor (serpin) superfamily and the inhibitor of urokinase and tissue plasminogen activators (uPA and tPA). PAI‐1 is implicated in the pathology of fibrosis in different organs, either excessive or deficient contributions leading to the development of fibrosis diseases development [9]. It is also known that PAI‐1 is mainly involved in the mechanism of thrombosis. Our recent study has identified that PAI‐1 levels were increased in the plasma of a FeCl_3_‐induced mouse carotid artery thrombosis model under chronic stress [10]. Except for that, PAI‐1 functions extend to various cell functions. For instance, it can inhibit cell migration by blocking integrin alpha V beta 3 binding to vitronectin and vascular smooth muscle cell apoptosis [11, 12]. Drug targeting of PAI‐1 inhibited atherogenesis and metabolic dysfunction in a murine metabolic syndrome model [13]. However, the precise mechanisms involved in the pathogenesis of atherosclerotic diseases including AAA remain largely unknown.

Epigallocatechin‐3‐gallate (EGCG), a catechin monomer isolated from tea leaves, is the main bioactive component of tea polyphenols. So far, EGCG is most commonly used for anti‐tumor effects. EGCG can inhibit the activation of matrix metalloproteinase‐2 (MMP‐2) and reduce the invasion ability of tumor [14]. Except for that, EGCG has the potential to prevent neurodegenerative diseases by blocking the accumulation of fibrin [15]. EGCG also participates in osteoclast differentiation through NF‐κB [16]. Moreover, EGCG has protective effects in cardiovascular disease, such as anti‐platelet aggregation, anti‐inflammatory, and anti‐oxidative stress [17, 18]. The findings from our previous studies demonstrate the inhibitory effects of EGCG on smooth muscle cell invasion and intimal hyperplasia in a rat carotid artery injury model [19, 20]. It has been suggested that EGCG attenuates AAA progression in a rat model [21]. However, the specific mechanism of EGCG in preventing AAA is still unclear.

Thus, we conducted this study to investigate the involvement of PAI‐1 in CaCl_2_‐induced AAA formation in mice and the potential protective effect of EGCG on AAA formation. PAI‐1 deficiency (PAI‐1^−/−^) mice and wild‐type (PAI‐1^+/+^) mice were used to build AAA models and PAI‐1^+/+^ mice with or without oral administration of EGCG. The results verified our hypothesis that EGCG can prevent the development of AAA by upregulating the expression of PAI‐1 and anti‐inflammatory, anti‐oxidative stress, anti‐proteolysis, and anti‐apoptosis. With regard to the vascular smooth muscle cells (VSMC), which play an important role in AAA formation, we validated our hypothesis by using hydrogen peroxide (H_2_O_2_)‐induced cell injury models and PAI‐1 silenced or overexpressed VSMCs treated with or without EGCG in vitro.

## 2 Materials and Methods

### 2.1 Experimental Animals

Males wild‐type [PAI‐1^+/+^, C57BL/6J] were obtained from the Animal Experimental Center of Yanbian University. PAI‐1 deficient (PAI‐1^−/−^, C57BL/6J) were purchased from Shanghai Biomodel Organism Science & Technology Development Co. Ltd., Shanghai, China and bred to generate for using animal experiments. Nine‐week‐old WT (PAI‐1^+/+^) and PAI‐1^−/−^ mice used in the experiments weighed 20–25 g. Mice were provided a standard diet and tap water ad libitum and housed four per cage under standard conditions (50% ± 5% humidity, 23°C ± 1°C) with a 12 h light/dark cycle (dark beginning at 7:00 pm) in a viral pathogen‐free facility at the Animal Center of Yanbian University. The animal study protocol (No. YD20231120003) was approved by the Ethics Committee of Animal Research at Yanbian University Medical College. All animal experiments were performed in accordance with the guidelines of animal care of Yanbian University.

### 2.2 AAA Model

The experimental AAA model was described previously [22]. In brief, PAI‐1^+/+^ and PAI‐1^−/−^ mice were anesthetized with isoflurane (3%–5% in an induction chamber and between 0.5% and 2% for maintenance, depending on the procedure and its duration). The abdominal aorta was exposed between the left renal artery and the iliac bifurcation. Afterwards, the aorta was wrapped in gauze soaked with 1 mol/L CaCl_2_ (Solarbio, Beijing, China) for 30 min, changing to new gauze every 15 min (Figure 2A). The gauze was then removed and the intraperitoneal cavity was washed thoroughly three times using 0.9% saline. A gauze with 0.9% saline was used for the sham operation as the related control for PAI‐1^+/+^ and PAI‐1^−/−^ mice.

### 2.3 EGCG Treatment Experiment

PAI‐1^+/+^ mice were randomly divided into three groups: a control group, a AAA group, and an EGCG group. For the EGCG group, EGCG (MedChemExpress, Shanghai, China) solution was administered orally by gavage every day from 7 days (2.5 × 10^−2^ mg/g daily) before the induction of AAAs and continued for another 4 weeks until sacrifice. The control group and AAA group received tap water over the interval.

### 2.4 Tissue Collections for Analysis

Mice were euthanized with isoflurane. For biological evaluation, animals were perfused with 5 mL isotonic saline at physiological pressure, and then the subrenal arteries were isolated and kept in RNAlater solution (Invitrogen, CA, USA) or −80°C freezer [23]. For morphological studies, the subrenal arteries were isolated with the heart and scale to measure the maximum diameter of the arteries. After being immersed in 4% fixative solution (Solarbio) overnight (4°C) and dehydrated using graded sucrose, vessels were embedded in Tissue Tek optimal cutting temperature compound (Sakura, Tokyo, Japan) and stored at −80°C.

### 2.5 Morphometric and Immunohistological Analysis

In mice, 5‐μm‐thick cryosections at aortic aneurysm segments were prepared. Corresponding sections were stained through three dying methods. Hematoxylin and eosin (H&E) staining (Solarbio) was used to evaluate aortic wall thickness, Masson staining (Solarbio) was used to detect aortic fibrosis and collagen content, and Elastic Van Gieson (EVG) staining (Abcam, Cambridge, MA, UK) was performed to detect aortic fibrin rupture. For the analysis of elastin degradation, 5 cross‐sections of aortas in each artery were evaluated according to the aneurysm classification, and a scoring system between 1 to 4 was used (a score of 1 was defined as no elastin degradation, a score of 2 as mild elastin degradation, a score of 3 as severe elastin degradation and a score of 4 as aortic rupture) [23]. For analysis, Image J software was used to measure collagen fibers fields. For the evaluation of the elastin disruption degree, the 4–5 images of each vessel (100× magnification) were calculated, the elastin score and collagen deposition area, and averaged the value for each mouse.

### 2.6 Immunofluorescence

The frozen sections were incubated in 5% goat serum for 1 h after being fixed by Sodium citrate‐EDTA antigen repair solution (Beyotime, dilution 1:40). They were incubated at 4°C overnight with anti‐F4/80 Rat (1:100, CST#71299), Anti‐alpha‐SMA Ab (1:500, AF‐1032, Affinity), Anti‐PAI‐1 Ab (1:400, 66261‐1‐Ig, Proteintech), and Anti‐CD31 Ab (1:400, 28083‐1‐AP, Proteintech), followed by the second antibodies Goat Anti‐Rat IgG H&L (1:500, ab6840, Abcam), Goat Anti‐Rabbit IgG H&L (1:500, ab150078), and Goat Anti‐Mouse IgG (H + L) (1:500, K1204, APExBIO) 2‐h incubation in the shade. DAPI (Beyotime) was added on top of the cover glass slides, which were visualized using an Evos microscope (Invitrogen, CA, USA) [24]. The high‐power field of view was randomly selected, and positive cells were observed as green fluorescence. For the quantification of positive cell staining, we evaluated five images of each aortic section using a 40× objective, and we calculated the numbers of F4/80^+^ cells and averaged the numbers for each mouse. For the statistical analysis of immune co‐localization, we referred to a previous study [25].

### 2.7 RNA Extraction and Gene Expression Assay

RNA was harvested from tissue and cultured cells with TRIzol (Invitrogen). mRNA was reverse‐transcribed to cDNA with a reverse transcriptase kit (ZOMANBIO, Beijing, China). Then cDNAs were applied to a real‐time quantitative polymerase chain reaction (qPCR) using an ABI 7300 real‐time PCR system (Applied Biosystems, Foster City, CA) with primers specific for matrix metalloprotease‐2 (*MMP‐2*), *MMP‐9*, gp91^phox^, intercellular adhesion molecule‐1 (*ICAM‐1*), monocyte chemotaxis protein‐1 (*MCP‐1*), vascular cell adhesion molecule‐1 (*VCAM‐1*), interleukin‐1β (*IL‐1β*), glyceraldehyde 3‐phosphate dehydrogenase (*GAPDH*), *Collagen I*, *and Collagen III*. The Ct values were normalized to GAPDH, and the relative gene expression was calculated using the −ΔΔCt method [26]. All experiments were performed in triplicate. The primer sequences are listed in Table 1.

**TABLE 1:** Primer sequences.

Genes	Forward primers	Reverse primers
gp91^phox^	ACTTTCCATAAGATGGTAGCTTGG	GATTCACACACCACTCAACG
ICAM‐1	CCCCGCAGGTCCAATTC	CCAGAGCGGCAGAGCAA
VCAM‐1	ACAAAACGATCGCTCAAATCG	GGTGACTCGCAGCCCGTA
MMP‐2	CCCCATGAAGCCTTGTTTACC	TTGTAGGAGGTGCCCTGGAA
MMP‐9	CCAGACGCTCTTCGA GAACC	GTTATAGAAGTGGCGGTTGT
Collagen I	AGGCGAAGGCAACAGTCG	GTTCCGGYGTGACTCGTGC
Collagen III	AGGTTCTCCTGGTGCTGCT	GGATGCCCACTTGTTCCAT
PAI‐1	TCTGGGAAAGGGTTCACTTTACC	GACACGCCATAGGGAGAGAAG
GAPDH	ATGTGTCCGTCGTGGATCTGA	ATGCCTGCTTCACCACCTTCT

Abbreviations: GAPDH, glyceraldehyde‐3‐phosphate dehydrogenase; ICAM‐1, intercellular adhesion molecule‐1; MMP‐2, matrix metalloproteinase‐2; MMP‐9, matrix metalloproteinase‐9; PAI‐1, plasminogen activator inhibitor‐1; VACM‐1, vascular cell adhesion molecule‐1.

### 2.8 Immunoblotting Assay

The mouse aortic VSMCs and the aortic tissues were lysed in RIPA with protease and phosphatase inhibitors (100:1:1) for 30 min on ice to extract total protein [27]. The supernatants were collected after 10 min centrifugation (13 000 rpm). The concentration of each protein was measured by the BCA protein assay kit (Solarbio). Then the protein samples were heated at 95°C for 10 min mixed with SDS‐PAGE. Cooling to room temperature, the denatured proteins were equally loaded and separated by SDS‐PAGE. Proteins were transferred to PVDF membranes. Then 5% bovine serum albumin (BSA) or milk in TBST (Tris‐Buffered Saline and Tween 20) was used to block the membranes. The membranes were incubated at 4°C overnight with primary antibodies against PAI‐1 (6626‐1‐Ig, Proteintech, Chicago, USA), transforming growth factor‐beta (TGF‐β, CST#3711), p‐Smad2/3 (CST#8828) cleaved‐caspase‐8 (C‐casp8, CST#8592), GAPDH (CST#5174, five from Cell Signaling Technology), collagen I (ab260043, Abcam), collagen III (ab184993, Abcam) and gp91^phox^ (Cat: 611415, BD Transduction Laboratories) at a 1:1000 dilution. Membranes were treated with the HRP‐conjugated secondary antibody (1:5000 dilution) for 2 h and washed three times in TBST at room temperature [11]. Chemiluminescent substrates (Millipore) were used to visualize immunoblot bands. The signal intensities were analyzed and calculated by software (Azure Biosystems, CA, USA).

### 2.9 Gelatin Zymography

Equal amounts of total protein (30 μg) extracted from the aortas were mixed with sodium dodecyl sulfate (SDS) sample buffer with 5× non‐reducing sample buffer and loaded onto a 10% SDS‐polyacrylamide gel containing gelatin as described [28]. After electrophoresis, the gels were washed twice for 30 min each time with washing buffer, and then incubated in the incubation buffer at 37°C overnight. Following staining with staining solution for 30 min to 1 h, the gels were incubated with destaining solution until bands can clearly be seen. The above assay was mentioned in the manufacturer's instructions of Abcam Gelatin Zymography Protocol. (Abcam, Cambridge, UK).

### 2.10 NADPH Oxidase Activity Assay

Superoxide (O_2_^−^) production extracted from homogenates of aortic tissue was assessed using a lucigenin‐based enhanced chemiluminescence assay, following the described [29]. Lucigenin (concentration 5 μmol/L) was used to reduce artificial O_2_^−^ production due to REDOX cycling. The final concentrations of protein were 100 and 5 μmol/L before the measurement of chemiluminescence. All of the assays were performed in triplicate.

### 2.11 Immunosorbent Assay

Plasma samples taken from the supernatant of left ventricular blood with heparin were used for the enzyme‐linked immunosorbent assay (ELISAs) [30]. The levels of interleukin‐1 beta (IL‐1β), Tumor necrosis factor‐alpha (TNF‐α), mouse plasma, and cell supernatant PAI‐1 were measured using the ELISA kit (MLBIO, Shanghai, China) according to the manufacturer's instructions.

### 2.12 Cell Culture

Mouse aortic VSMCs (also MOVAS, Fuheng Biotechnology, Shanghai, China) and Mouse aortic endothelial cells (also ECs, Pricella Biotechnology, Wuhan, China) were cultured in Dulbecco's modified Eagle's medium (DMEM, Viva Cell, Shanghai, China) supplemented with 200 μg/mL G418 (Geneticin, Beyotime, Shanghai, China) and 10% fetal bovine serum (FBS, Pricella, Hubei, China) in an incubator at 37°C with 5% CO_2_ [30]. All cells were used for subsequent experiments.

### 2.13 Gene Transfection

PAI‐1 gene silencing and overexpression were performed as previously described [31]. The VSMCs were seeded in 6‐well plates (3 × 10^5^cells/well) 12–24 h prior to transfection. Short interfering RNA against PAI‐1 (siPAI‐1) and negative control (siNC) were used to silence the PAI‐1 gene (Table 2), as well as pcDNA3.1(+)‐PAI‐1 and negative control (plNC; all from GenePharma, Shanghai, China) were used to overexpress the PAI‐1 gene. The VSMCs were grown on 60‐mm dishes until 80% confluence with the use of a Lipofectamine 3000 Transfection Kit following the manufacturer's instructions. The cells were cultured at 37°C for 48 h, and the levels of targeted protein and gene were evaluated by western blotting, PCR, or ELISA. Transfected cells were also used for the next cellular experiments.

**TABLE 2:** siRNA sequences used for gene transfection.

Genes	Forward primers	Reverse primers
Serpine1‐mus‐381	GGCAGAUCCAAGAUGCUAUTT	AUAGCAUCUUGGAUCUGCCTT
Serpine1‐mus‐732	GCCAAUGGAAGACUCCUUUTT	AAAGGAGUCUUCCAUUGGCTT
Serpine1‐mus‐1124	GCCGACUUCACAAGUCUUUTT	AAAGACUUGUGAAGUCGGCTT

### 2.14 Cell Treatment

Tiplaxtinin (PAI‐039, APExBIO, Houston, USA) known as a PAI‐1 inhibitor was applied to VSMC experiments at indicated concentration (10 μM). Following transfection with siPAI‐1 or plPAI‐1, respectively, the VSMCs were pretreated with and without EGCG (20 μM) for 30 min, and then were cultured in H_2_O_2_ at indicated concentration (0, 400 μM) for 24 h. TGF‐β receptor inhibitor (SB 431542, APExBIO) was also used in the VSMC experiments at indicated concentration (10 μM). The results were subjected to western blotting analysis.

### 2.15 Cell Apoptosis Assay

Mouse aortic VSMCs were seeded in 12‐well plates (2 × 10^4^cells/well) and allowed to adhere overnight. After transfection, the cells were cultured in serum‐free DMEM medium containing H_2_O_2_ and EGCG at indicated concentrations for 12 h and then were subjected to a terminal deoxynucleotidyl transferase‐mediated dUTP‐biotin nick‐end labeling (TUNEL) assay with the use of an In Situ Cell Death detection kit (Beyotime) according to the manufacturer's instructions [32]. The apoptotic TUNEL^+^ cells were calculated over 3 randomly selected fields of each well and averaged for the expression.

### 2.16 Network Pharmacology

A thorough search was conducted on the OMIM (https://omim.org/), NCBI (https://www.ncbi.nlm.nih.gov/), and Genecards (https://www.genecards.org/) databases to identify a total of 4118 *Mus Musculus* targets linked with AAA after removing duplicates. The TCMSP (http://tcmspnw.com/), SwissTargetPrediction (http://swisstargetprediction.ch/), Pharmmapper(http://www.lilab‐ecust.cn/pharmmapper/), and SuperPred (https://prediction.charite.de/) databases were sequentially searched for EGCG‐related targets, and 162 targets were obtained after removing duplicates. Two target networks were generated for AAA and EGCG using Cytoscape 3.9.1 software and its stringApp. Key nodes with degree values exceeding the average were identified by analyzing the intersection nodes of the two networks. The David Platform performed enrichment analysis on the major nodes of AAA and EGCG, including Kyoto Encyclopedia of Genes and Genomes (KEGG) and Gene Ontology (GO). The data was visually represented using bar charts or bubble charts.

### 2.17 Statistical Analysis

Data are expressed as mean ± SEM. All statistical analyses were performed using prism (GraphPad Prism 8.0.2; GraphPad Software Inc., La Jolla, CA). Student's *t* tests (for comparison between 2 groups) or 1‐way ANOVA (for comparisons of ≥ 3 groups) followed by Tukey's post hoc tests were used for the statistical analyses. After the distribution status of the test data was determined, the data were subjected to the statistical analyses. The AAA morphological analyses were evaluated by two observers in a blind manner, and the values they obtained were averaged. A value of *p* < 0.05 was considered statistically significant.

## 3 Results

### 3.1 Network Pharmacology Analysis for Target Molecules

**FIGURE 1:**
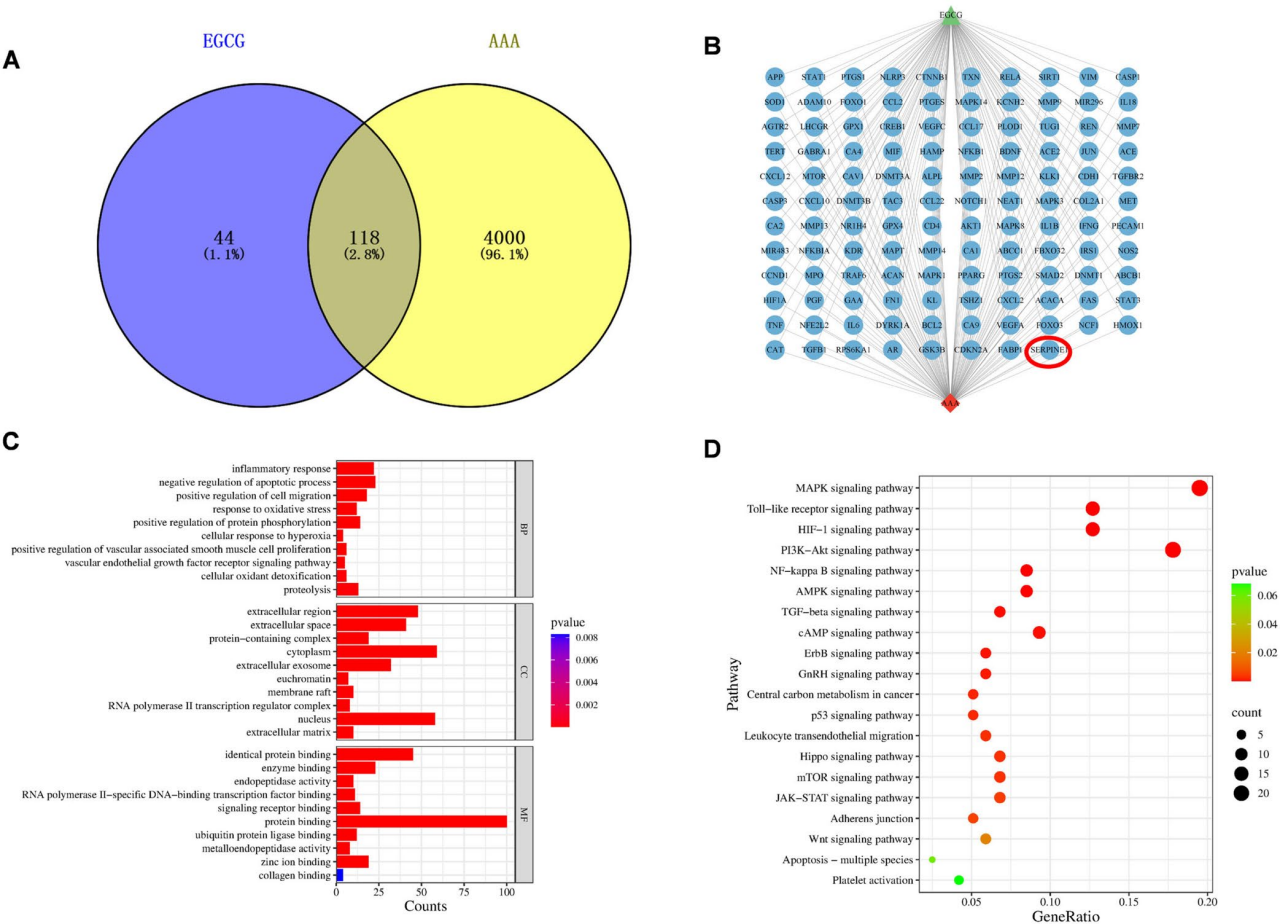
Network pharmacology analysis was used for target Molecules of EGCG‐mediated prevention of AAA. (A, B) The process of screening potential and core targets of the EGCG‐mediated inhibitory effect on AAA. (C, D) The KEGG and GO enrichment analyses revealed the inhibitory effect of EGCG on AAA at the molecular and cellular levels. All KEGG and GO analyses were performed using the David database (https://david.ncifcrf.gov/).

To investigate the potential molecular targets of EGCG‐mediated protection on AAA formation, a network pharmacology analysis was conducted. A total of 162 genes associated with EGCG were identified using the SwissTargetPrediction database, while 4118 genes related to AAA were retrieved from the GeneCards database. Subsequently, a Venn diagram was constructed using the Bioinformatics platform, revealing 118 overlapping targets (Figure 1A). These 118 common targets were imported into the STRING database, and a protein–protein interaction (PPI) network was constructed using Cytoscape 3.9.1 software. We found that PAI‐1 was among the target genes (Figure 1B).

In order to systematically explore the potential mechanisms of EGCG‐mediated anti‐AAA formation, we performed a GO analysis. We found that EGCG may affect inflammation, apoptosis, and oxidative stress production (Figure 1C). Moreover, we used KEGG pathway analysis to identify the signaling pathways associated with EGCG‐mediated prevention of AAA. We observed 154 statistically significant activated pathways (e.g., TGF‐β signaling pathway) of the 20 relevant pathways identified (Figure 1D). These findings suggest that EGCG might protect against AAA formation through the modulation of the TGF‐β signaling pathway, providing a theoretical basis for further molecular mechanistic exploration and therapeutic targets.

### 3.2 CaCl_2_ ‐Mediated AAA Induction Lowered PAI‐1 Expression and Increased Oxidative Stress

**FIGURE 2:**
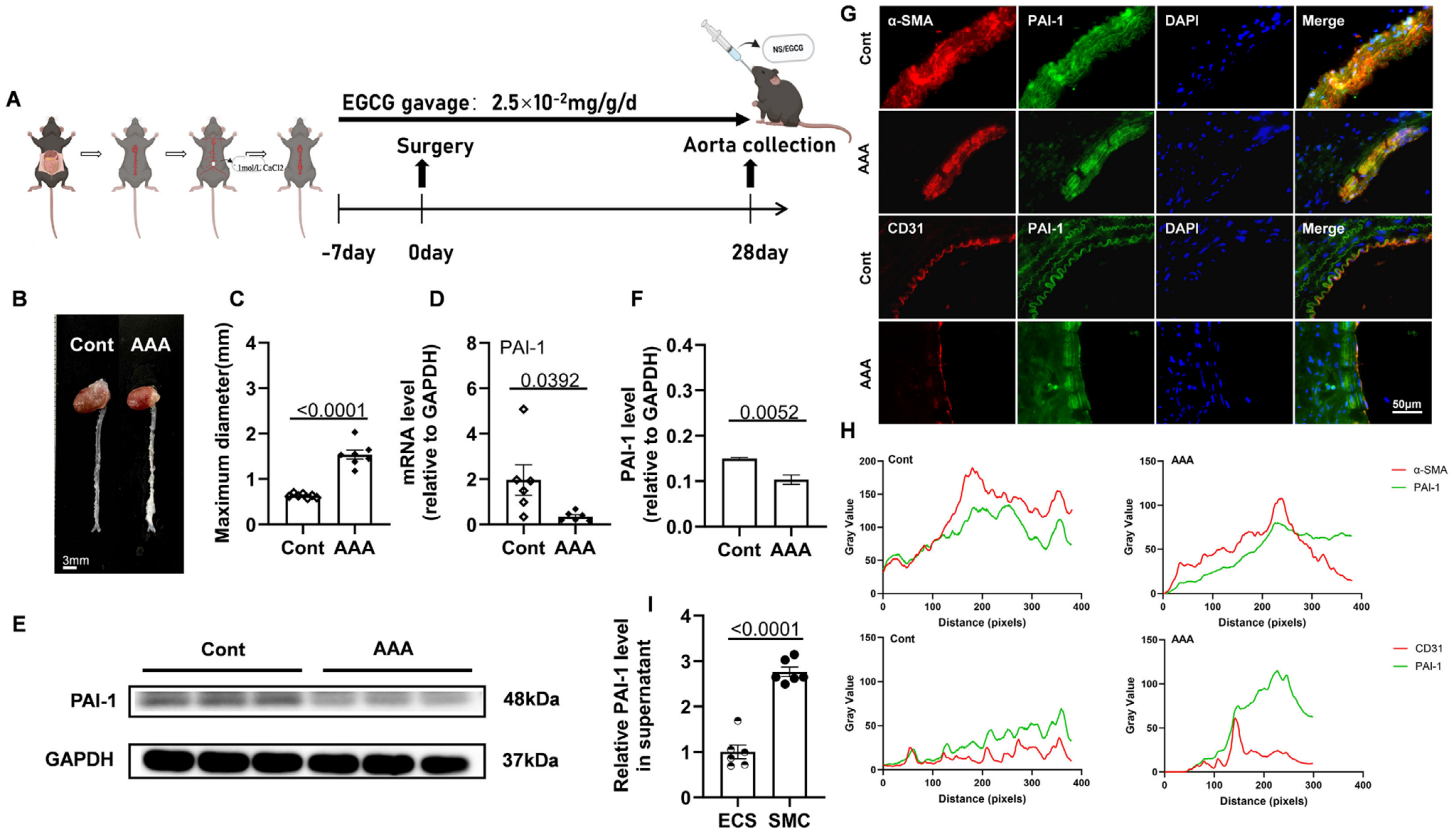
The four‐week CaCl_2_‐induced AAA model decreased abdominal arterial PAI‐1 expression. (A) Elementary diagram of the mouse AAA surgery and sampling procedures at the indicated time points under EGCG treatment. (B, C) Representative photographs and quantitative data of the aortic maximum diameter in two groups (*n* = 7/group). (E, F) Representative Western Blotting image and quantitative data of PAI‐1 protein in two experimental groups (*n* = 3/group). (D) qPCR data show PAI‐1 mRNA levels in both groups (*n* = 6/group). Scale bar: 3 μm. (G, H) Representative image and quantitative data of immunostaining of PAI‐1 and α‐SMA or CD31 in two groups (DAPI, blue; α‐SMA or CD31, red; PAI‐1, green). Scale bar: 50 μm. (I) Representative relative PAI‐1 level in supernatant in two cell types (*n* = 6/group). Data are mean ± SEM. Statistical significance was assessed by Student *t* test for C, D, F, and I. Cont indicates NaCl control and AAA indicates CaCl_2_ model.

CaCl_2_‐induced AAA model was often used in human aorta aneurysmic peripheral artery disease [23]. AAA is characterized by dilation or rupture of the aorta. After four weeks of AAA inducement of CaCl_2_ (Figure 2A), the abdominal aortas of mice in the AAA group gained severe aneurysm. Compared to the control group, the maximum diameter of aortas in the AAA group was increased by 2.4‐fold (Figure 2B,C). The PCR results showed that the mRNA level of PAI‐1 was lower in the aortic lesions compared to the normal tissues (Figure 2D). Likewise, the immunoblotting assay demonstrated a decline in the PAI‐1 protein in AAA tissue (Figure 2E,F). ELISA showed that the level of plasma PAI‐1 also decreased in models (Figure S1A). To identify the cell sources of PAI‐1, we performed double immunofluorescence using anti‐PAI‐1 and anti‐α‐SMA or anti‐PAI‐1 and anti‐CD31 antibodies, respectively. We found that the double staining signal was greater in the medial smooth muscle cell area than in the endothelial cell area of the control mice, and the double staining signal was dramatically reduced in the lesions of the AAA (Figure 2G,H). In the in vitro experiment, we observed that the substantial amount of PAI‐1 protein was in the SMC‐cultured medium (Figure 2I).

### 3.3 PAI‐1 Deficiency Promotes Inflammation, Oxidative Stress, and Proteolysis Leading to Aggravations of AAA Formation

**FIGURE 3:**
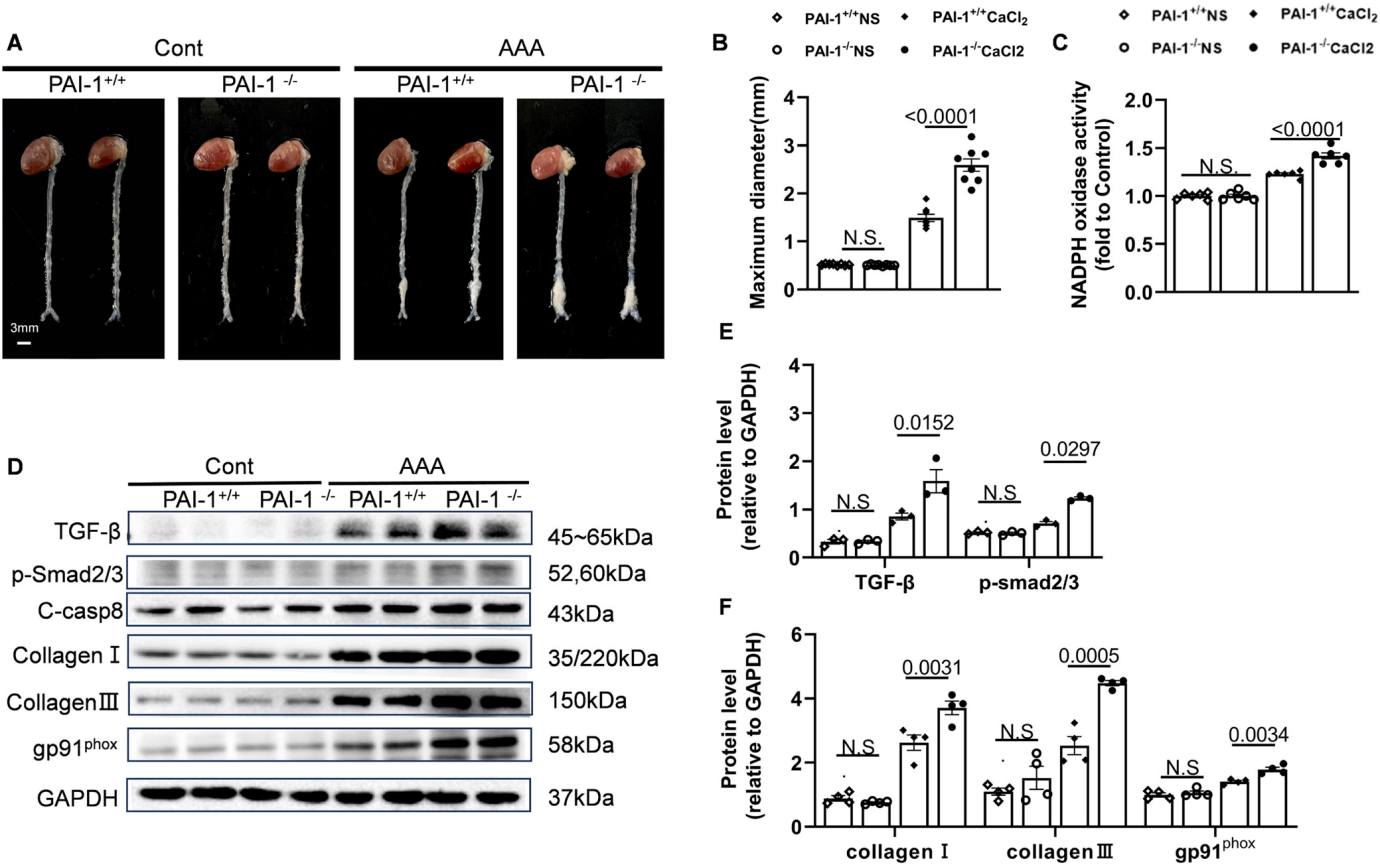
PAI‐1 deficiency aggravated the CaCl_2_‐induced AAA formation, oxidative stress, and the targeted protein levels in the AAA tissues. (A, B) Representative photographs and data of the aortic maximum diameter in PAI‐1^+/+^ and PAI‐1^−/−^mice treated with NaCl or CaCl_2_ (*n* = 8/group). (C) Quantitative data show the NADPH (nicotinamide adenine dinucleotide phosphate) oxidase activity of aortic tissues in the four experimental groups (*n* = 6/group). (D–F) Representative Western Blotting images and quantitative data for the levels of TGF‐β (transforming growth factor‐β), p‐Smad2/3, C‐casp8 (cleaved‐caspase 8), collagen I, collagen III, and gp91^phox^ proteins in 4 groups (*n* = 3/group). Data are mean ± SEM. Statistical significance was assessed by 1‐way ANOVA for B, C, E, and F. Scale bar: 3 μm. N.S. indicates no significance.

**FIGURE 4:**
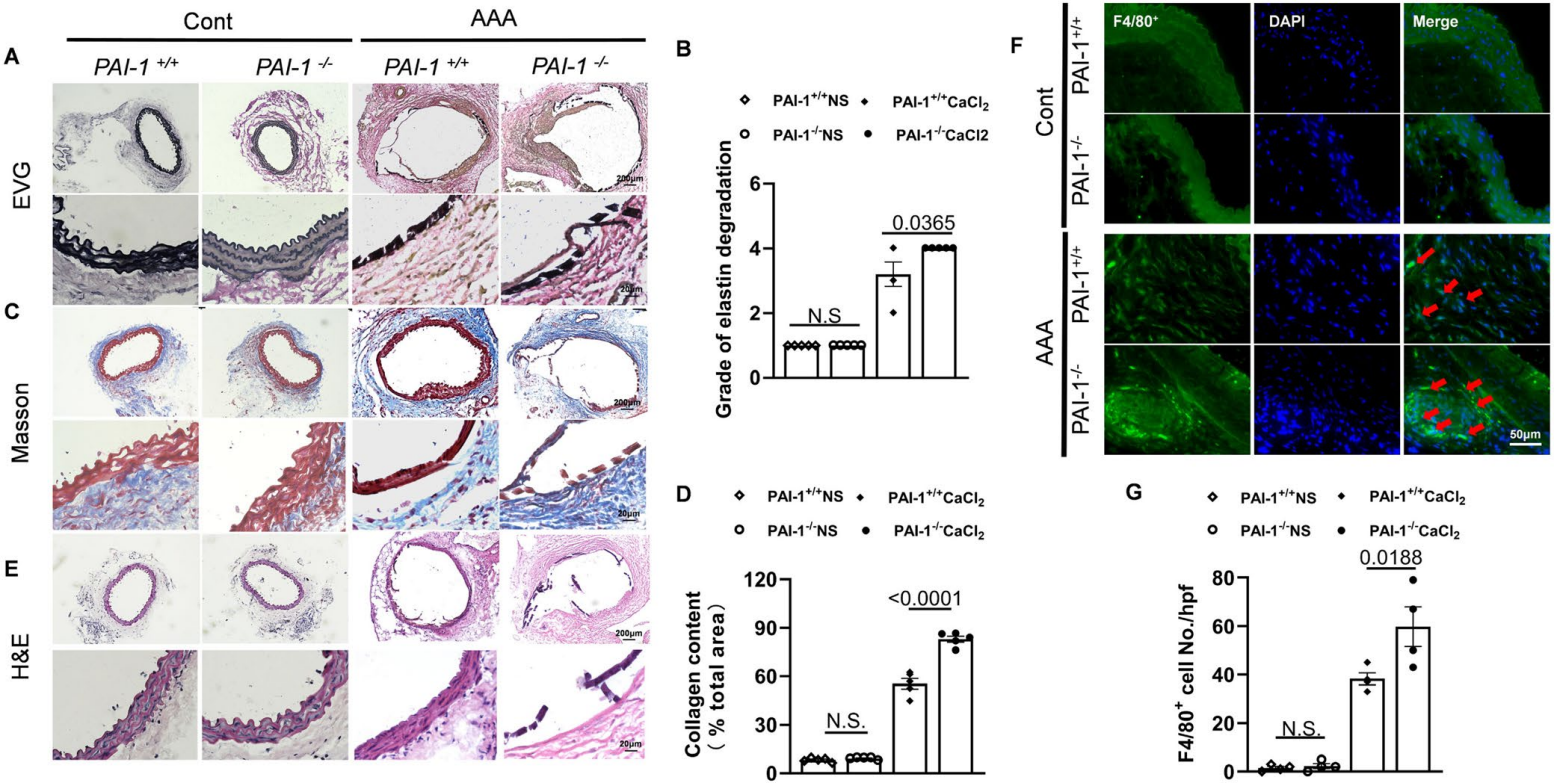
PAI‐1 deficiency deteriorated the histopathological changes and inflammatory cell infiltration. (A, B) Representative EVG (Elastic Van Gieson) staining images and quantitative data for elastin lamina disruption in four groups (*n* = 5/group). (C, D) Representative Masson staining images and quantitative data for the collagen content in four groups (*n* = 5/group). (E) Representative images of H&E (hematoxylin and eosin) staining for four groups. Scale bars: 200 and 20 μm. (F, G) Representative immunostaining images and quantitative data for F4/80^+^ macrophages in four groups (*n* = 4/group). Scale bars: 50 μm. Data are mean ± SEM. Statistical significance was assessed by 1‐way ANOVA for B, D, and G.

As shown in Figure 3A,B, the maximum diameter of the AAA lesion in PAI‐1^−/−^ mice was increased compared with PAI‐1^+/+^ mice. While NADPH oxidase activity was higher by 1.2‐fold in PAI‐1^−/−^‐CaCl_2_ mice than that of PAI‐1^+/+^‐CaCl_2_ mice (Figure 3C). PAI‐1^−/−^ resulted in an increase in the levels of gp91^phox^, TGF‐β, p‐Smad2/3, collagen I, and collagen III which were apparently enhanced in the AAA lesions of the PAI‐1^−/−^‐CaCl_2_ group (Figure 3D–F). Through Figure 4, it is easy to obtain that PAI‐1 disruption exacerbated elastin degradation, increased collagen content in the AAA lesion area, and F4/80^+^ macrophage infiltration. Moreover, the targeted genes [gp91^phox^, intercellular adhesion molecule‐1 (ICAM‐1), vascular cell adhesion molecule‐1 (VCAM‐1), collagen I and III, matrix metalloproteinase (MMP‐2), and (MMP‐9)] were higher in the AAA PAI‐1^−/−^ mice compared with the AAA PAI‐1^+/+^ mice (Figure S2A–D). Likewise, the activity of MMP‐2 and MMP‐9 had the same variation tendency with significant (Figure S3A,B). And the levels of TNF‐α and IL‐1β in plasma also augmented in AAA PAI‐1^−/−^ mice (Figure S1B).

### 3.4 EGCG Increased PAI‐1 Expression and Lowered Inflammation, Oxidative Stress, Proteolysis, and Apoptosis, Leading to Prevention of CaCl_2_ ‐Induced AAA

**FIGURE 5:**
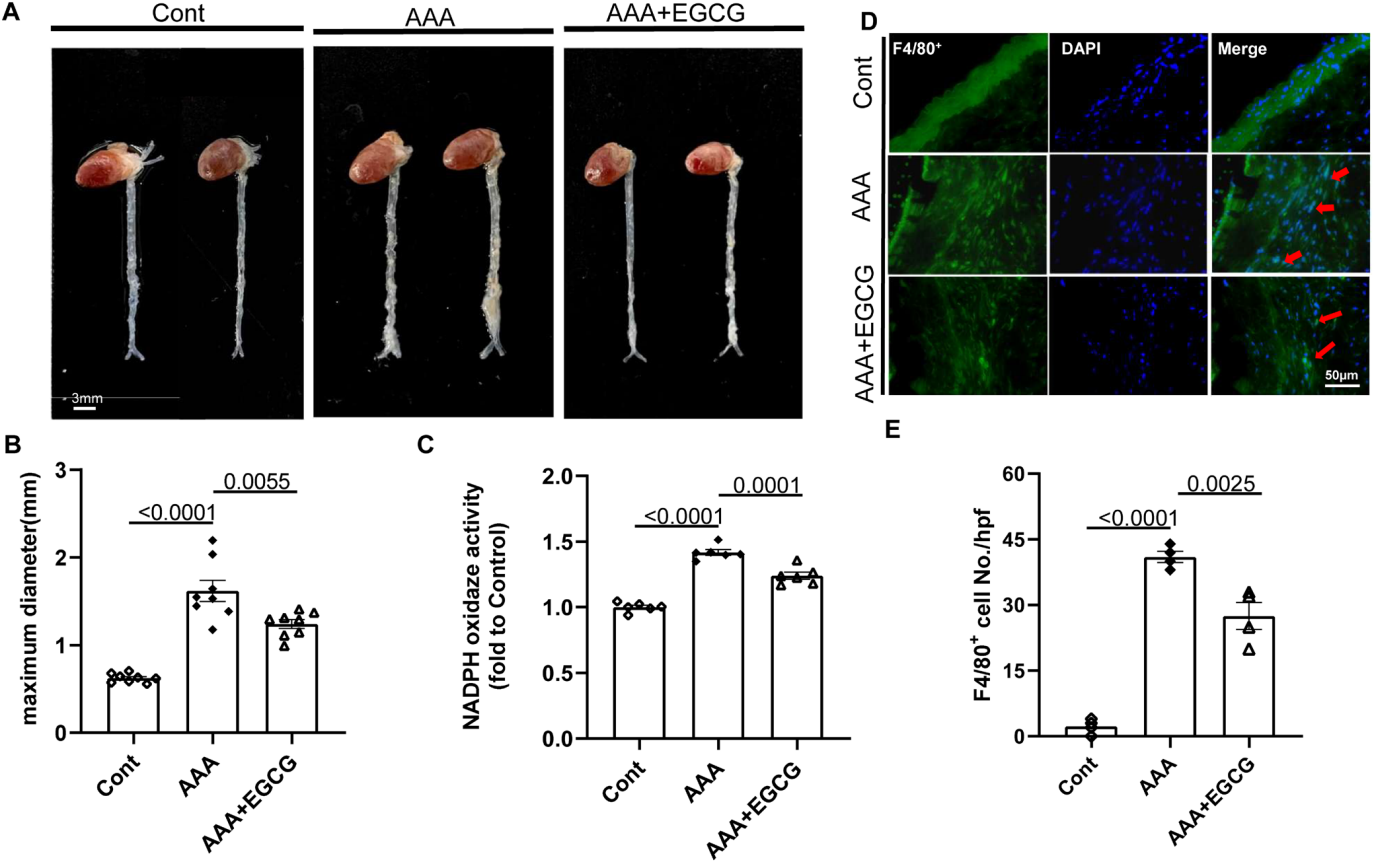
EGCG mitigated the CaCl_2_‐induced AAA formation, oxidative stress, and inflammatory cell infiltration. (A, B) Representative photographs and quantitative data of the aortic maximum diameter in the control group, AAA group, and AAA + EGCG group (*n* = 8/group). Scale bar: 3 μm. (C) Quantitative data show the NADPH oxidase activity of aortic tissues in three groups (*n* = 6/group). (D, E) Representative immunostaining images and quantitative data for F4/80^+^ macrophages in three groups (*n* = 4/group). Scale bar: 50 μm. Data are mean ± SEM. Statistical significance was assessed by 1‐way ANOVA for B, C, and E. AAA + EGCG indicates CaCl_2_‐induced AAA mice were treated with EGCG gavage.

**FIGURE 6:**
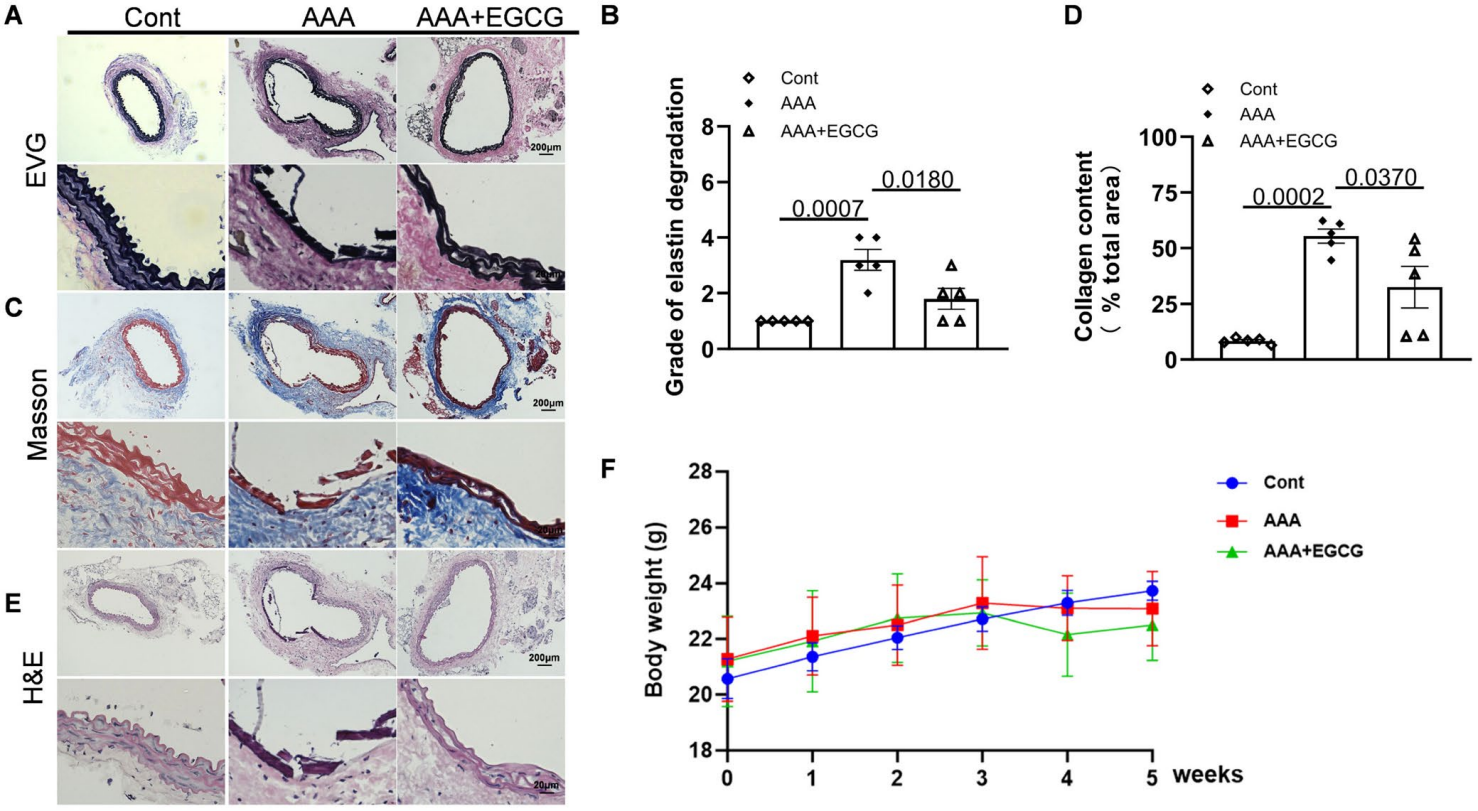
EGCG alleviated the histopathological changes. (A, B) Representative EVG staining images and quantitative data for the media elastic lamina disruption in three groups (*n* = 5/group). (C, D) Representative Masson staining images and quantitative data for the collagen contents in three groups (*n* = 5/group). (E) Representative H&E staining images in three groups. Scale bars: 200 and 20 μm. (F) Representative body weight (BW) in four groups (*n* = 7/group). Data are mean ± SEM. Statistical significance was assessed by 1‐way ANOVA for B and D, 2‐way ANOVA for F.

**FIGURE 7:**
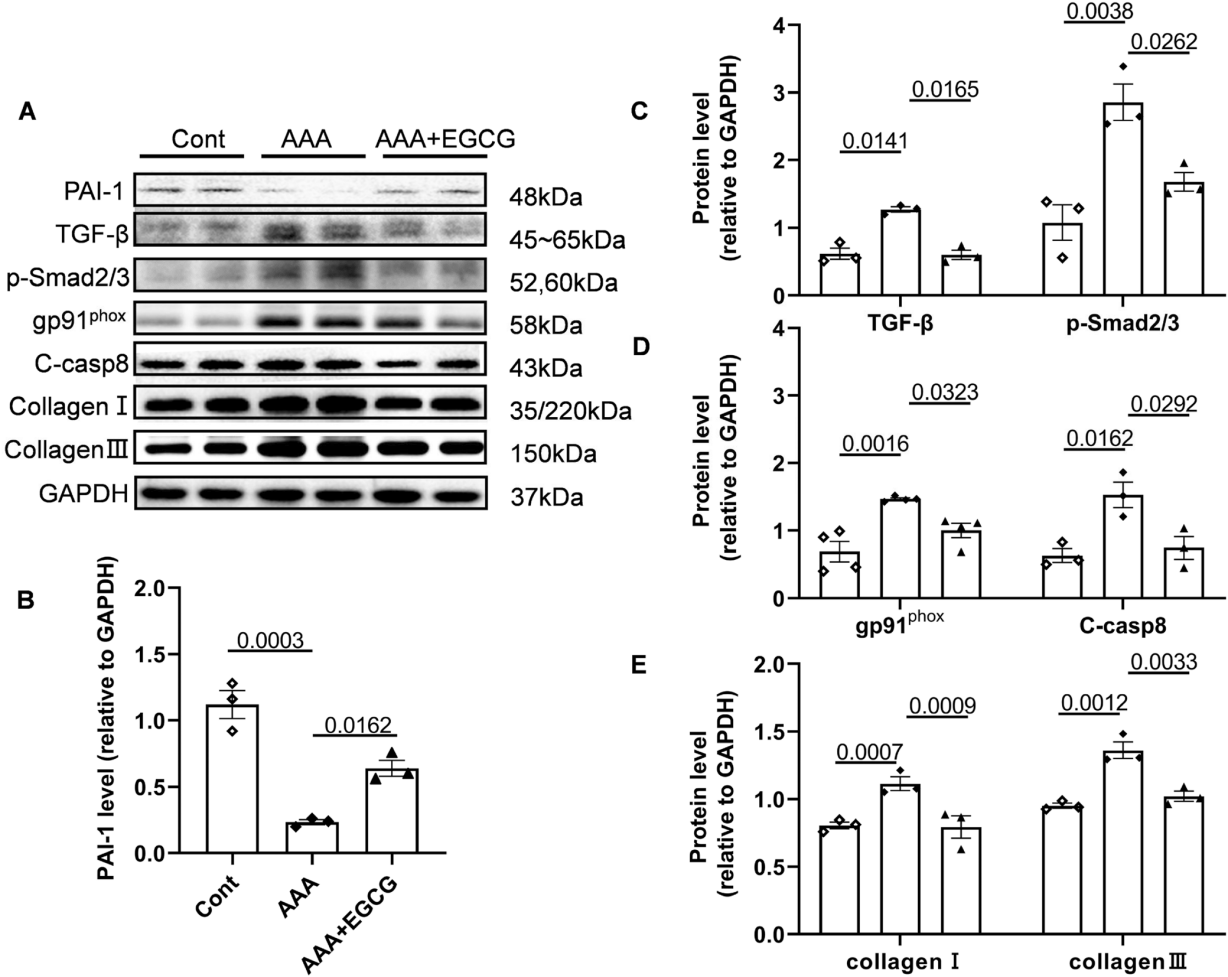
EGCG reduced the protein expression in aortic tissues induced by CaCl_2_. (A–E) Representative Western Blotting images and quantitative data for the levels of PAI‐1, TGF‐β, p‐Smad2/3, gp91^phox^, C‐casp8, collagen I, and collagen III proteins in three groups (*n* = 3/group). Data are mean ± SEM. Statistical significance was assessed by 1‐way ANOVA.

Each mouse in the EGCG group received a dose of 2.5 × 10^−2^ mg/kg daily of EGCG solution that was administered orally from 7 days before the induction of AAAs and continued for another 4 weeks (Figure 2A). As shown by the representative photos and quantitative data in Figure 5A,B, EGCG markedly reduced the expansion of the aorta and maximum diameter compared to AAA PAI‐1^+/+^ mice. We observed that EGCG can lessen NADPH oxidase activity (Figure 5C). The main pathological changes of AAA include macrophage infiltration, rupture of elastic fibers in the blood vessel wall, and expansion of the aortic wall as H&E staining showed were relieved by EGCG treatment (Figures 5D,E and 6E). While elastin fiber degradation is a prominent feature, EVG staining revealed that the elastic lamellae lost their curvature and became flat, and the fragmentation of the elastic lamellae appeared in AAA PAI‐1^+/+^ mice. EGCG treatment significantly mitigated the degradation of elastin (Figure 6A,B). Masson staining revealed a significant reduction in collagen content in the AAA mice treated by EGCG compared to that of the PAI‐1^+/+^ mice (Figure 6C,D). The aforementioned findings indicated that EGCG has the potential to effectively mitigate the progression and intensity of aneurysms in mice induced by CaCl_2_. The expression of PAI‐1 was upregulated after treatment with EGCG (Figures 7A,B and S1C). The harmful alterations in the levels of gp91^phox^, TGF‐β, p‐Smad2/3, C‐casp8, collagen I and III proteins were observed in AAA PAI‐1^+/+^ mice (Figure 7C–E), and these changes were rectified by EGCG. Representative gelatin zymography images of MMP‐2 and MMP‐9 activity, which are shown in Figure S3C,D, demonstrated that EGCG significantly reduced both gelatinolytic activities. In addition, TNF‐α and IL‐1β levels in plasma also decreased when treated with EGCG (Figure S1D). Thus, these observations suggest that EGCG‐mediated anti‐inflammation, anti‐oxidative stress, and anti‐apoptosis effects might contribute to vascular protective actions. However, there was no significant difference in the BW between the three experimental groups (Figure 6F).

### 3.5 EGCG Alleviated H_2_O_2_ ‐Induced VSMCs Apoptosis Aggravated by PAI‐1 Inhibition and the Effects Were in Line With PAI‐1 Overexpression

**FIGURE 8:**
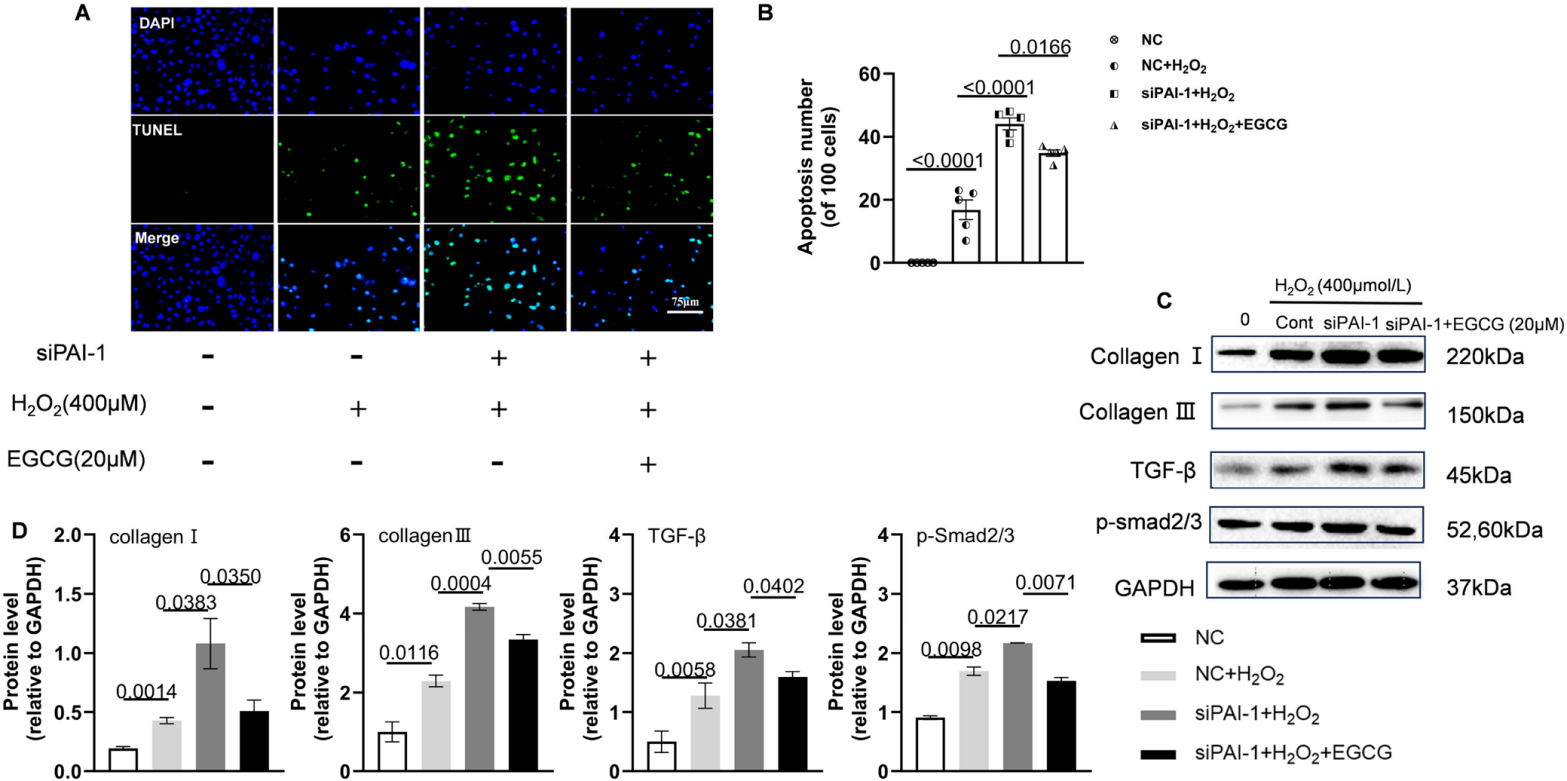
EGCG alleviated PAI‐1 silenced MAVSMCs apoptosis in response to H_2_O_2_. (A, B) Representative TUNEL staining images and quantitative data for the apoptotic cells in four groups (*n* = 5/group). Scale bar: 75 μm. (C, D) Representative Western Blotting images and quantitative data for the levels of collagen I, collagen III, TGF‐β, and p‐Smad2/3 proteins in four groups (*n* = 3/group). Data are mean ± SEM. Statistical significance was assessed by 1‐way ANOVA for B and D. NC indicates negative control. siPAI‐1 indicates PAI‐1 silencing.

**FIGURE 9:**
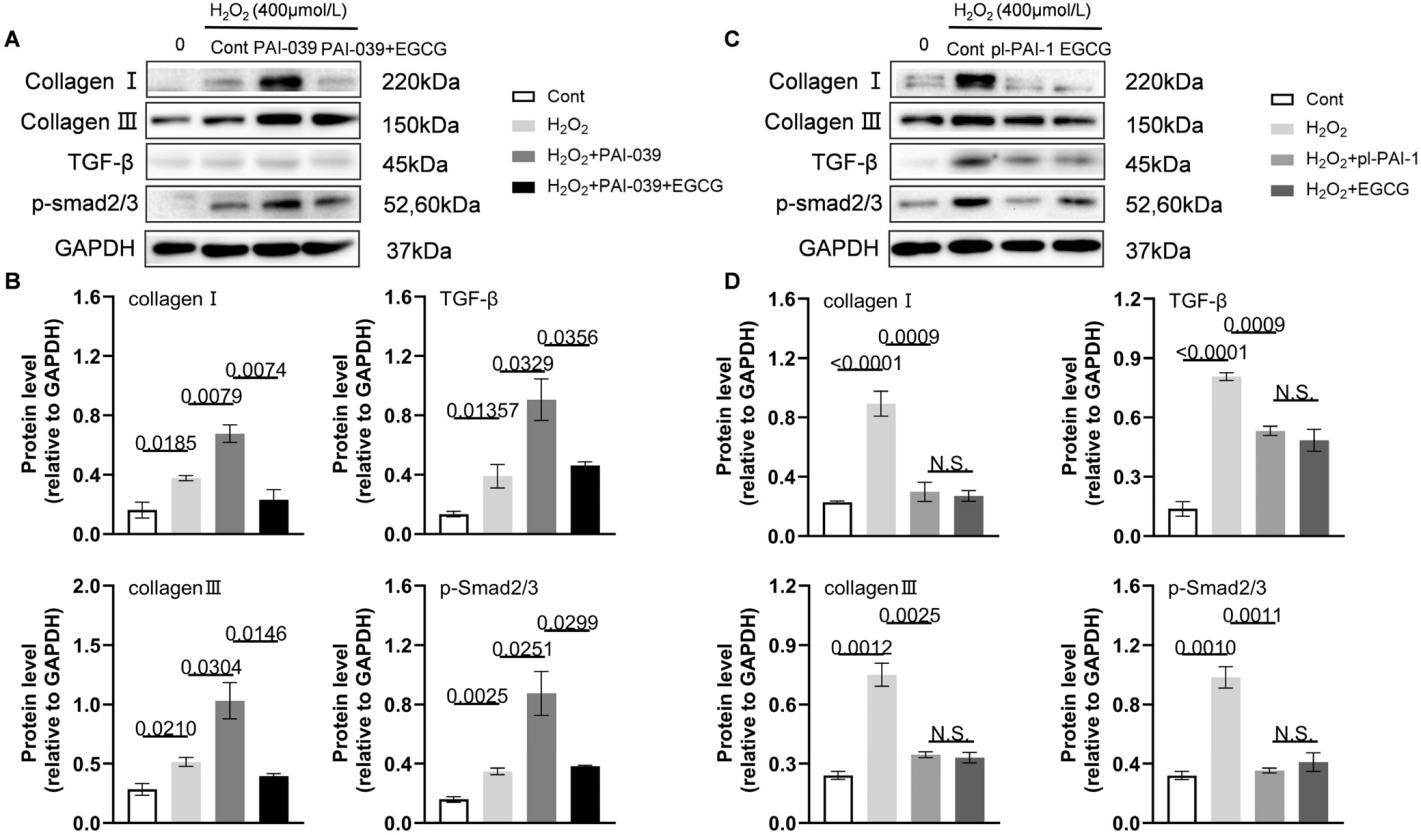
EGCG decreased H_2_O_2_‐induced extracellular matrix protein turnover‐related targeted proteins expressions in MAVSMCs treated with PAI‐1 inhibitor and plPAI‐1. (A, B) Representative Western Blotting images and quantitative data for the levels of collagen I, collagen III, TGF‐β, and p‐Smad2/3 proteins in Cont, H_2_O_2_, H_2_O_2_ + PAI‐039, H_2_O_2_ + PAI‐039 + EGCG groups (*n* = 3/group). (C, D) Representative Western Blotting images and quantitative data for the levels of collagen I, collagen III, TGF‐β, and p‐Smad2/3 proteins in Cont, H_2_O_2_, H_2_O_2_ + pl‐PAI‐1, H_2_O_2_ + EGCG groups (*n* = 3/group). Data are mean ± SEM. Statistical significance was assessed by 1‐way ANOVA for B and D. NC indicates negative control. PAI‐039 indicates PAI‐1 inhibitor. pl‐PAI‐1 indicates PAI‐1 overexpression.

**FIGURE 10:**
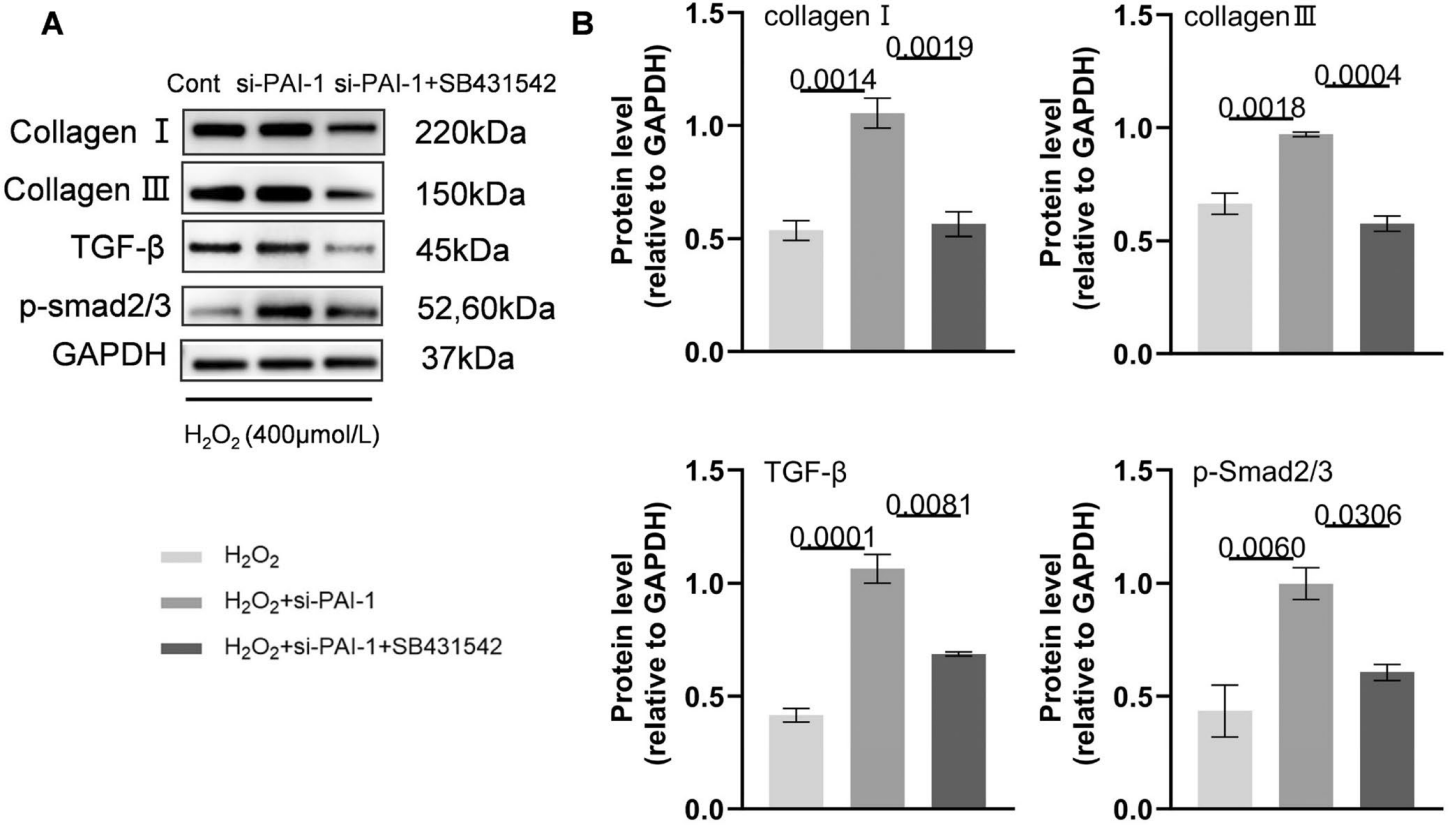
TGF‐β receptor inhibitor inhibited H_2_O_2_‐induced its downstream molecule expressions in VSMCs transfected with siPAI‐1. (A, B) Representative Western Blotting images and quantitative data for the levels of collagen I, collagen III, TGF‐β, and p‐Smad2/3 proteins in H_2_O_2_, H_2_O_2_ + siPAI‐1, H_2_O_2_ + si‐PAI‐1 + SB431542 groups (*n* = 3/group). Data are mean ± SEM. Statistical significance was assessed by 1‐way ANOVA for B.

To verify the roles of PAI‐1, we conducted a series of in vitro experiments. At first, the PAI‐1 gene was silenced by siRNA transfection to VSMCs. As shown in Figure S4, the green fluorescent was observed in VSMCs, and the results of the qPCR and Western Blotting proved that PAI‐1 was knocked down significantly in PAI‐1 siRNA 2, which was chosen to do following experiments. After that, to simulate AAA formation in vitro, we built an H_2_O_2_‐induced cell damage model. TUNEL staining showed that PAI‐1 silencing aggravated H_2_O_2_‐induced VSMC apoptosis, and this effect was rectified by EGCG (Figure 8A,B). Similar to the data in vivo, the expression of TGF‐β, p‐Smad2/3, collagen I, and III in the VSMCs stimulated with H_2_O_2_ was increased. On this basis, genetic and pharmacological inhibitions of PAI‐1 increased the expression of four targeted proteins, and these effects were dramatically reversed by EGCG treatment (Figures 8C,D and 9A,B). On the contrary, the PAI‐1 overexpression plasmid DNA was transfected into the VSMCs. The green fluorescent was observed in VSMCs, and the results of Western Blotting and ELISA of cell supernatant proved that PAI‐1 was overexpressed (Figure S5A–D). Based on H_2_O_2_ induced cell damage model, PAI‐1 overexpression alleviated the expression of those four proteins, and the effects were similar to EGCG treatment (Figure 9C,D). Interestingly, the TGF‐β receptor inhibitor (SB 431542) lowered the levels of the H_2_O_2_‐induced collagen I, collagen III, TGF‐β, and p‐Smad2/3 proteins in VSMCs transfected with siPAI‐1 (Figure 10). Collectively, these observations suggest that EGCG‐mediated vasculoprotection might be due to the induction of PAI‐1 and reduction of TGF‐β, p‐Smad2/3, collagen I and III, as well as cell apoptosis.

## 4 Discussion

The above‐mentioned experiments focused on the role of PAI‐1 in mechanisms of AAA formation induced by CaCl_2_ and how EGCG plays a protective function in AAA development. The most significant finding of this study is that mice lacking the PAI‐1 gene exacerbated AAA development. At the morphological and molecular levels, PAI‐1 deficiency was shown to have harmful alterations as follows: (i) an increase in the maximum diameter and collagen content and enhancement of elastin degradation in AAA lesions; (ii) NADPH oxidase oxidative stress production; and (iii) increasing of inflammation‐(ICAM‐1, VCAM‐1, TNF‐α, and IL‐1β), apoptosis‐(C‐casp8), proteolysis (MMP‐2/‐9), and extracellular matrix (ECM) turnover (TGF‐β, p‐Smad2/3, collagen I and collagen III)‐related genes and/or proteins in the aortic tissues and plasma of AAA mice. In vitro, PAI‐1 overexpression or silencing and PAI‐1 inhibitor (PAI‐039), respectively, decreased and increased the levels of the targeted proteins (TGF‐β, p‐Smad2/3, collagen I and collagen III) and apoptosis in VSMCs under oxidative stress conditions, providing evidence and a mechanistic explanation of the negative regulation of PAI‐1 on TGF‐β/Smad2/3 signaling activation in CaCl_2_‐induced AAA formation. While EGCG loading can reverse the pathogenesis of AAA in the following aspects and upregulated the expression of PAI‐1 as well as ameliorated targeted PAI‐1^−/−^‐induced inflammation, oxidative stress, ECM turnover, and apoptosis‐related protein harmful changes. In vitro, our results demonstrated that EGCG prevented apoptosis in VSMCs under oxidative stress conditions, indicating that EGCG prevented AAA through upregulating PAI‐1 protein, anti‐inflammation, anti‐apoptosis, and anti‐oxidative stress. The mechanism underlying EGCG‐mediated vasculoprotective actions in mice under our experimental AAA induction conditions is schematically presented in Figure S6.

PAI‐1, a member of the serine protease inhibitor (SERPINE) family, is well‐known in thrombosis and fibrosis [9]. In early (1998 and 2008), two previous studies showed overexpression can prevent angiotensin II infusion‐induced murine AAA development and guinea pig‐to‐rat aortic xenograft AAA development and rupture [33, 34], whereas there was no evidence of whether PAI‐1 deletion deteriorates AAA formation. In this study, CaCl_2_‐AAA induction stress resulted in a reduction in the levels of PAI‐1 gene and protein in plasma and/or the aortic tissues of PAI‐1^+/+^ AAA mice. we have shown that the maximum diameter of AAA lesion in PAI‐1^−/−^ mice was increased compared with PAI‐1^+/+^ mice, suggesting that PAI‐1 acts as a key mediator of the AAA formation process in mice under our experimental conditions.

The most characteristic feature behind AAA formation involves the degradation of ECM [35]. The main components of the ECM are collagen and elastin [36]. PAI‐1 can elevate the expressions of basement membrane proteins (collagen and fibronectin) and proteolytic enzymes (MMP‐2, MMP‐9) during lung injury and fibrosis [37]. In our study, our results showed the expression of collagen I and III mRNAs was increased in the AAA lesions of PAI‐1^−/−^ mice. It is consistent with the data of Masson staining assay of PAI‐1^−/−^ mice that had accumulated collagen in the aortic AAA lesions compared to the PAI‐1^+/+^ mice. Accumulating evidence suggests that MMPs, particularly MMP‐2 and ‐9, are the main enzymes responsible for the degradation of ECM in AAA initiation and progression [23, 38]. We have shown that the levels of MMP‐2 and MMP‐9 mRNAs and their gelatinolytic activities in the aneurysmic lesions were increased in the PAI‐1^−/−^ mice compared to those of PAI‐1^+/+^ mice. TGF‐β signaling has been significantly implicated in the pathogenesis of aneurysm, such as MMP‐dependent proteolysis, vascular inflammation, and SMC growth and apoptosis [39]. A well‐established MDA‐MB‐231 breast cancer model showed that the MMP‐9 upregulation was attenuated by selective inhibition of the TGF‐βRI/Smad3 pathway in breast cancer metastasis [40]. It has been demonstrated that CaCl_2_ treatment induces the upregulation of TGF‐β which contributed to the increase in Smad3 phosphorylation [41]. It has been demonstrated that PAI‐1 regulated TGF‐β signaling in cardiac fibrosis [42]. Our present findings demonstrate the levels of TGF‐β and p‐Smad2/3 as well as collagen type I and III were significantly increased in PAI‐1^−/−^ mice. Moreover, PAI‐1^−/−^ resulted in an increase in the levels of oxidative stress (NADPH activity and gp91^phox^)‐, apoptosis (C‐casp8)‐, inflammation‐(ICAM‐1, VCAM‐1, TNF‐α, and IL‐1β)‐related genes and/or proteins. Taken together, PAI‐1 deficiency appears to promote CaCl_2_‐induced AAA formation via the negative modulations of inflammation, apoptosis, proteolysis, and oxidative stress that were mediated by TGF‐β/Smad2/3 signaling activation in mice under our experimental condition. However, it should be noted that there is a discrepancy between elevated MMP levels and collagen accumulation in our CaCl_2_‐induced AAA model. A recent review article highlighted that there were unbalances between collagen production and degradation, as well as the enzymes (MMPs and cysteinyl cathepsins) and their endogenous inhibitors (tissue inhibitors of MMPs and cystatin C) that contribute to the cardiovasculo‐renal tissue fibrosis [43]. Several studies demonstrated that MMPs‐ and cathepsins‐mediated extracellular matrix protein degradation products can promote vascular and inflammatory cell function and collagen expression in vitro and in vivo [44, 45, 46]. Moreover, it was reported that mutated and spliced collagen was resistant to protease degradation [47]. These findings raise the possibility that the harmful alterations (collagen mutation/splicing, matrix protein degradation products, and an unbalance between matrix production and proteolytic activity as well as enzymes and endogenous inhibitors, etc.) might contribute to the collagen accumulation of the aneurysmic adventitia in mice under our experimental conditions.

EGCG is the predominant polyphenol found in green tea. As is widely acknowledged, EGCG has been found to possess a diverse array of therapeutic properties, which include anti‐atherosclerosis, anti‐cardiac hypertrophy, anti‐myocardial infarction, anti‐diabetes, anti‐inflammatory, and antioxidant [48]. Several studies demonstrated that dietary polyphenols reduce the viability of adipocytes, suppress adipocyte differentiation, stimulate lipolysis and fatty acid β‐oxidation, and reduce inflammation [49, 50, 51]. Two previous laboratory studies reported that oral administration of green tea polyphenol prevented AAA progression [21, 52]. These studies have only examined morphological aspects. Our observations here show that EGCG loading suppressed aorta diameter enlargement, elastic laminal disruption, and collagen accumulation associated with the reduction of the aneurysmic lesion MMP‐2 and MMP‐9 expression and activities. Interestingly, EGCG pretreatment increased PAI‐1 protein levels in plasma and aortic AAA lesions in PAI‐1^+/+^ mice. Because PAI‐1 participates in cellular function and human pathobiology, we proposed that PAI‐1 functions as an important mediator of the EGCG‐mediated vascular response. Previously, it was reported that EGCG can suppress harmful reactive oxygen species and ECM component collagen type I production by the modulation of connective tissue growth factor/extracellular regulated protein kinase‐1/2 and nuclear factor kappa‐B signaling, leading to the mitigation of systemic sclerosis [53]. EGCG pretreatment reversed elevated plasma NADPH oxidase activity and inflammatory cytokine (TNF‐α and IL‐1β) levels, and macrophage infiltration into the aortic lesions. EGCG also ameliorated the harmful changes in the levels of ECM turnover‐related proteins (collagen I and III, TGF‐β, and p‐Smad2/3) and apoptosis‐related (NADPH oxidase component protein (gp91phox)) in the aneurysmic lesions of PAI‐1^+/+^ mice. In the mouse aortic VSMC experiment yielded the same conclusion was reached in which EGCG inhibited VSMC apoptosis as well as these four protein changes in response to H_2_O_2_. Thus, the upregulation of PAI‐1 by EGCG treatment could represent a common mechanism in the protection of vascular tissues from CaCl_2_‐AAA induction stress via the reduction of oxidative stress production, inflammation, proteolysis, and apoptosis.

Study limitations should be considered. First, PAI‐1 overexpression and its pharmacological inhibitor could not be used to further explore the exact role of PAI‐1 in AAA formation in mice under the present experimental conditions. And we also could provide the direct evidence regarding TGF‐β/Smad2/3‐dependent MMP‐2 and MMP‐9 expressions. Second, we did not satisfactorily explain a discrepancy between elevated MMP levels and collagen accumulation in our AAA model. Third, since only male mice were used, the possible influence of gender‐related factors such as menstruation and sex hormones could not be examined. Finally, with regard to the modeling of aneurysms, angiotensin II‐induced hypertensive AAAs are more similar to the mechanisms of human AAAs. That AAA model was not applied to test the study hypothesis. Further investigations are necessary to address these issues.

In conclusion, our findings have demonstrated that the levels of PAI‐1 gene and protein were decreased in plasma and/or the aortic tissues of CaCl_2_‐induced AAA mice. PAI‐1 deletion deteriorated CaCl_2_‐induced AAA formation by the increase of TNF‐α and IL‐1β secretions, leading to increased macrophage recruitment, which induces inflammation, oxidative stress, and ECM degradation, providing a potential management target against AAA. Based on the EGCG‐mediated upregulation of PAI‐1, the investigated PAI‐1 deficiency‐mediated morphological and molecular harmful changes were mitigated. Therefore, to the best of our knowledge, the present study is the first to provide evidence of PAI‐1 deficiency‐mediated AAA aggravation and EGCG‐mediated anti‐AAA formation by the induction of PAI‐1 expression, providing a possible pharmacological molecular target for AAA management.

## Author Contributions

**Mantong Zhao:** conceptualization (lead); data curation (lead); investigation (lead); methodology (lead); project administration (lead); resources (lead); software (lead); writing – original draft (lead). **Lina Hu:** data curation (equal); methodology (supporting); supervision (supporting); validation (supporting); writing – review and editing (equal). **Zhuo Lin:** data curation (supporting); methodology (supporting); investigation (supporting); **Xueling Yue:** methodology (supporting); software (supporting); visualization (supporting). **Xintong Zheng:** methodology (supporting); visualization (supporting). **Meiling Piao:** data curation (equal); methodology (supporting). **Xianglan Jin:** data curation (supporting); writing – review and editing (supporting). **Limei Piao:** visualization (supporting); writing – review and editing (supporting). **Rihua Cui:** visualization (supporting); writing – review and editing (Supporting). **Meilan Liu:** data curation (supporting); writing – review and editing (supporting). **Xian Wu Cheng:** conceptualization (lead); data curation (supporting); project administration (lead); resources (lead); supervision (lead); validation (lead); visualization (lead); writing – review and editing (lead).

## Consent

The authors have nothing to report.

## Conflicts of Interest

The authors declare no conflicts of interest.

## Supporting information


Data S1.

## Data Availability

The data underlying this article will be shared on reasonable request to the corresponding author.

## References

[1] K. Takemoto , M. Nakamura , M. Sakuraya , et al., “Infected Thoracoabdominal Aortic Aneurysm Related to an Implanted Long‐Term Arterial Catheter for Chemotherapy: A Case Report,” Journal of Medical Case Reports 15 (2021): 81, 10.1186/s13256-021-02661-4. 33610163 PMC7897388

[2] D. K. Owens , K. W. Davidson , A. H. Krist , et al., “Screening for Abdominal Aortic Aneurysm,” JAMA 322, no. 22 (2019): 2211, 10.1001/jama.2019.18928. 31821437

[3] R. F. Gillum , “Epidemiology of Aortic Aneurysm in the United States,” Journal of Clinical Epidemiology 48 (1995): 1289–1298. 7490591 10.1016/0895-4356(95)00045-3

[4] H. W. Kniemeyer , T. Kessler , P. U. Reber , H. B. Ris , H. Hakki , and M. K. Widmer , “Treatment of Ruptured Abdominal Aortic Aneurysm, a Permanent Challenge or a Waste of Resources? Prediction of Outcome Using a Multi‐Organ‐Dysfunction Score,” European Journal of Vascular and Endovascular Surgery: The Official Journal of the European Society for Vascular Surgery 19 (2000): 190–196, 10.1053/ejvs.1999.0980. 10727370

[5] J. R. Baman and M. K. Eskandari , “What Is an Abdominal Aortic Aneurysm?,” Journal of the American Medical Association 328 (2022): 2280, 10.1001/jama.2022.18638. 36511924

[6] J. Toczek , J. L. Meadows , and M. M. Sadeghi , “Novel Molecular Imaging Approaches to Abdominal Aortic Aneurysm Risk Stratification,” Circulation. Cardiovascular Imaging 9 (2016): e003023, 10.1161/CIRCIMAGING.115.003023. 26763279 PMC4714781

[7] F. A. M. V. I. Hellenthal , W. A. Buurman , W. K. W. H. Wodzig , and G. W. H. Schurink , “Biomarkers of Abdominal Aortic Aneurysm Progression. Part 2: Inflammation,” Nature Reviews Cardiology 6 (2009): 543–552, 10.1038/nrcardio.2009.102. 19546866

[8] M. R. Alexander and G. K. Owens , “Epigenetic Control of Smooth Muscle Cell Differentiation and Phenotypic Switching in Vascular Development and Disease,” Annual Review of Physiology 74 (2012): 13–40, 10.1146/annurev-physiol-012110-142315. 22017177

[9] H. Kaneko , T. Anzai , M. Morisawa , et al., “Resveratrol Prevents the Development of Abdominal Aortic Aneurysm Through Attenuation of Inflammation, Oxidative Stress, and Neovascularization,” Atherosclerosis 217 (2011): 350–357, 10.1016/j.atherosclerosis.2011.03.042. 21530968

[10] A. K. Ghosh and D. E. Vaughan , “PAI‐1 in Tissue Fibrosis,” Journal of Cellular Physiology 227 (2012): 493–507, 10.1002/jcp.22783. 21465481 PMC3204398

[11] S. Xu , L. Piao , Y. Wan , et al., “CTSS Modulates Stress‐Related Carotid Artery Thrombosis in a Mouse FeCl_3_ Model,” Arteriosclerosis, Thrombosis, and Vascular Biology 43 (2023): e238–e253, 10.1161/ATVBAHA.122.318455. 37128920

[12] S. Stefansson and D. A. Lawrence , “The Serpin PAI‐1 Inhibits Cell Migration by Blocking Integrin Alpha V Beta 3 Binding to Vitronectin,” Nature 383, no. 6599 (1996): 441–443, 10.1038/383441a0. 8837777

[13] Y. Chen , R. J. Kelm , R. C. Budd , B. E. Sobel , and D. J. Schneider , “Inhibition of Apoptosis and Caspase‐3 in Vascular Smooth Muscle Cells by Plasminogen Activator Inhibitor Type‐1,” Journal of Cellular Biochemistry 92 (2004): 178–188, 10.1002/jcb.20058. 15095413

[14] H. B. Khoukaz , Y. Ji , D. J. Braet , et al., “Drug Targeting of Plasminogen Activator Inhibitor‐1 Inhibits Metabolic Dysfunction and Atherosclerosis in a Murine Model of Metabolic Syndrome,” Arteriosclerosis, Thrombosis, and Vascular Biology 40 (2020): 1479–1490, 10.1161/atvbaha.119.313775. 32268785 PMC7255962

[15] L. Sartor , E. Pezzato , M. Donà , et al., “Prostate Carcinoma and Green Tea: (‐)Epigallocatechin‐3‐Gallate Inhibits Inflammation‐Triggered MMP‐2 Activation and Invasion in Murine TRAMP Model,” International Journal of Cancer 112 (2004): 823–829, 10.1002/ijc.20496. 15386368

[16] M. Pervin , K. Unno , T. Ohishi , H. Tanabe , N. Miyoshi , and Y. Nakamura , “Beneficial Effects of Green Tea Catechins on Neurodegenerative Diseases,” Molecules (Basel, Switzerland) 23, no. 6 (2018): 1297, 10.3390/molecules23061297. 29843466 PMC6099654

[17] R.‐W. Lin , C.‐H. Chen , Y.‐H. Wang , et al., “(‐)‐Epigallocatechin Gallate Inhibition of Osteoclastic Differentiation via NF‐kappaB,” Biochemical and Biophysical Research Communications 379 (2009): 1033–1037, 10.1016/j.bbrc.2009.01.007. 19150340

[18] H. J. Joo , J.‐Y. Park , S. J. Hong , et al., “Anti‐Platelet Effects of Epigallocatechin‐3‐Gallate in Addition to the Concomitant Aspirin, Clopidogrel or Ticagrelor Treatment,” Korean Journal of Internal Medicine 33 (2018): 522–531, 10.3904/kjim.2016.228. 29050464 PMC5943656

[19] L. Basiricò , P. Morera , D. Dipasquale , et al., “(−)‐Epigallocatechin‐3‐Gallate and Hydroxytyrosol Improved Antioxidative and Anti‐Inflammatory Responses in Bovine Mammary Epithelial Cells,” Animal: An International Journal of Animal Bioscience 13 (2019): 2847–2856, 10.1017/S1751731119001356. 31182175

[20] X. W. Cheng , M. Kuzuya , T. Sasaki , et al., “Green Tea Catechins Inhibit Neointimal Hyperplasia in a Rat Carotid Arterial Injury Model by TIMP‐2 Overexpression,” Cardiovascular Research 62 (2004): 594–602, 10.1016/j.cardiores.2004.01.023. 15158152

[21] X. W. Cheng , M. Kuzuya , K. Nakamura , et al., “Mechanisms of the Inhibitory Effect of Epigallocatechin‐3‐Gallate on Cultured Human Vascular Smooth Muscle Cell Invasion,” Arteriosclerosis, Thrombosis, and Vascular Biology 25 (2005): 1864–1870, 10.1161/01.ATV.0000179675.49619.9b. 16051878

[22] S. Setozaki , K. Minakata , H. Masumoto , et al., “Prevention of Abdominal Aortic Aneurysm Progression by Oral Administration of Green Tea Polyphenol in a Rat Model,” Journal of Vascular Surgery 65, no. 6 (2017): 1803–1812, 10.1016/j.jvs.2016.06.003. 27473778

[23] Z. Lin , M. Zhao , X. Zhang , et al., “CD8^+^ T‐Cell Deficiency Protects Mice From Abdominal Aortic Aneurysm Formation in Response to Calcium Chloride2,” Journal of Hypertension 42 (2024): 1966–1975, 10.1097/hjh.0000000000003823. 39146540 PMC11451972

[24] J. Fang , S. Shu , H. Dong , et al., “Histone Deacetylase 6 Controls Cardiac Fibrosis and Remodelling Through the Modulation of TGF‐β1/Smad2/3 Signalling in Post‐Infarction Mice,” Journal of Cellular and Molecular Medicine 28 (2024): e70063, 10.1111/jcmm.70063. 39232846 PMC11374528

[25] A. Prashar , C. Bussi , A. Fearns , et al., “Lysosomes Drive the Piecemeal Removal of Mitochondrial Inner Membrane,” Nature 632 (2024): 1110–1117, 10.1038/s41586-024-07835-w. 39169179 PMC7616637

[26] Y. Wan , L. Piao , S. Xu , et al., “Cathepsin S Deficiency Improves Muscle Mass Loss and Dysfunction via the Modulation of Protein Metabolism in Mice Under Pathological Stress Conditions,” FASEB Journal 37 (2023): e23086, 10.1096/fj.202300395RRR. 37428652

[27] Y. Wan , L. Piao , S. Xu , et al., “Cathepsin S Activity Controls Chronic Stress‐Induced Muscle Atrophy and Dysfunction in Mice,” Cellular and Molecular Life Sciences 80 (2023): 254, 10.1007/s00018-023-04888-4. 37589754 PMC10435624

[28] H. Jiang , X. W. Cheng , G. P. Shi , et al., “Cathepsin K‐Mediated Notch1 Activation Contributes to Neovascularization in Response to Hypoxia,” Nature Communications 5 (2014): 3838, 10.1038/ncomms4838. 24894568

[29] X. Jin , X. Yue , Z. Huang , et al., “Cathepsin K Deficiency Prevented Stress‐Related Thrombosis in a Mouse FeCl(3) Model,” Cellular and Molecular Life Sciences 81 (2024): 205, 10.1007/s00018-024-05240-0. 38703204 PMC11069486

[30] X. Meng , Z. Huang , A. Inoue , et al., “Cathepsin K Activity Controls Cachexia‐Induced Muscle Atrophy via the Modulation of IRS1 Ubiquitination,” Journal of Cachexia, Sarcopenia and Muscle 13 (2022): 1197–1209, 10.1002/jcsm.12919. 35098692 PMC8978007

[31] M. Zhang , X. Yue , S. Xu , et al., “Dipeptidyl Peptidase‐4 Disturbs Adipocyte Differentiation via the Negative Regulation of the Glucagon‐Like Peptide‐1/Adiponectin‐Cathepsin K Axis in Mice Under Chronic Stress Conditions,” FASEB Journal 38 (2024): e23684, 10.1096/fj.202400158R. 38795334

[32] X. Yue , L. Piao , H. Wang , et al., “Cathepsin K Deficiency Prevented Kidney Damage and Dysfunction in Response to 5/6 Nephrectomy Injury in Mice With or Without Chronic Stress,” Hypertension 79 (2022): 1713–1723, 10.1161/hypertensionaha.122.19137. 35726642 PMC9278705

[33] E. Allaire , D. Hasenstab , R. D. Kenagy , B. Starcher , M. M. Clowes , and A. W. Clowes , “Prevention of Aneurysm Development and Rupture by Local Overexpression of Plasminogen Activator Inhibitor‐1,” Circulation 98 (1998): 249–255, 10.1161/01.cir.98.3.249. 9697825

[34] H. S. Qian , J. M. Gu , P. Liu , et al., “Overexpression of PAI‐1 Prevents the Development of Abdominal Aortic Aneurysm in Mice,” Gene Therapy 15 (2008): 224–232, 10.1038/sj.gt.3303069. 18033310

[35] H. Kuivaniemi , E. J. Ryer , J. R. Elmore , and G. Tromp , “Understanding the Pathogenesis of Abdominal Aortic Aneurysms,” Expert Review of Cardiovascular Therapy 13 (2015): 975–987, 10.1586/14779072.2015.1074861. 26308600 PMC4829576

[36] J. Xu and G. P. Shi , “Vascular Wall Extracellular Matrix Proteins and Vascular Diseases,” Biochimica et Biophysica Acta 1842 (2014): 2106–2119, 10.1016/j.bbadis.2014.07.008. 25045854 PMC4188798

[37] F. M. Moideen , M. P. Rahamathulla , R. Charavu , F. Alghofaili , M. Sha , and Y. P. Bhandary , “PAI‐1 Influences and Curcumin Destabilizes MMP‐2, MMP‐9 and Basement Membrane Proteins During Lung Injury and Fibrosis,” International Immunopharmacology 143 (2024): 113587, 10.1016/j.intimp.2024.113587. 39549545

[38] C. Qiu , Y. Li , L. Xiao , et al., “A Novel Rabbit Model of Abdominal Aortic Aneurysm: Construction and Evaluation,” Heliyon 9 (2023): e17279, 10.1016/j.heliyon.2023.e17279. 37389075 PMC10300360

[39] F. Gao , P. Chambon , S. Offermanns , et al., “Disruption of TGF‐β Signaling in Smooth Muscle Cell Prevents Elastase‐Induced Abdominal Aortic Aneurysm,” Biochemical and Biophysical Research Communications 454 (2014): 137–143, 10.1016/j.bbrc.2014.10.053. 25450370

[40] S. Kochumon , A. Al‐Sayyar , T. Jacob , et al., “TGF‐β and TNF‐α Interaction Promotes the Expression of MMP‐9 Through H3K36 Dimethylation: Implications in Breast Cancer Metastasis,” Frontiers in Immunology 15 (2024): 1430187, 10.3389/fimmu.2024.1430187. 39351229 PMC11439675

[41] X. Dai , J. Shen , N. P. Annam , et al., “SMAD3 Deficiency Promotes Vessel Wall Remodeling, Collagen Fiber Reorganization and Leukocyte Infiltration in an Inflammatory Abdominal Aortic Aneurysm Mouse Model,” Scientific Reports 5 (2015): 10180, 10.1038/srep10180. 25985281 PMC4434993

[42] P. Flevaris , S. S. Khan , M. Eren , et al., “Plasminogen Activator Inhibitor Type I Controls Cardiomyocyte Transforming Growth Factor‐β and Cardiac Fibrosis,” Circulation 136 (2017): 664–679, 10.1161/circulationaha.117.028145. 28588076 PMC5784400

[43] X. W. Cheng , M. Narisawa , H. Wang , and L. Piao , “Overview of Multifunctional Cysteinyl Cathepsins in Atherosclerosis‐Based Cardiovascular Disease: From Insights Into Molecular Functions to Clinical Implications,” Cell & Bioscience 13 (2023): 91, 10.1186/s13578-023-01040-4. 37202785 PMC10197855

[44] M. Yang , Y. Zhang , J. Pan , et al., “Cathepsin L Activity Controls Adipogenesis and Glucose Tolerance,” Nature Cell Biology 9 (2007): 970–977, 10.1038/ncb1623. 17643114

[45] Q. Yao , E. The , L. Ao , et al., “TLR4 Stimulation Promotes Human AVIC Fibrogenic Activity Through Upregulation of Neurotrophin 3 Production,” International Journal of Molecular Sciences 21 (2020): 1276, 10.3390/ijms21041276. 32074942 PMC7072994

[46] B. Wang , J. Sun , S. Kitamoto , et al., “Cathepsin S Controls Angiogenesis and Tumor Growth via Matrix‐Derived Angiogenic Factors,” Journal of Biological Chemistry 281 (2006): 6020–6029, 10.1074/jbc.M509134200. 16365041

[47] M. Ju , X. Bai , T. Zhang , et al., “Mutation Spectrum of COL1A1/COL1A2 Screening by High‐Resolution Melting Analysis of Chinese Patients With Osteogenesis Imperfecta,” Journal of Bone and Mineral Metabolism 38 (2020): 188–197, 10.1007/s00774-019-01039-3. 31414283

[48] Q. Y. Eng , P. V. Thanikachalam , and S. Ramamurthy , “Molecular Understanding of Epigallocatechin Gallate (EGCG) in Cardiovascular and Metabolic Diseases,” Journal of Ethnopharmacology 210 (2018): 296–310, 10.1016/j.jep.2017.08.035. 28864169

[49] S. A. Zarasvand , V. Haley‐Zitlin , O. Oladosu , et al., “Assessing Anti‐Adipogenic Effects of Mango Leaf Tea and Mangiferin Within Cultured Adipocytes,” Diseases 11 (2023): 70, 10.3390/diseases11020070. 37218883 PMC10204365

[50] E. S. Kao , M. Y. Yang , C. H. Hung , C. N. Huang , and C. J. Wang , “Polyphenolic Extract From *Hibiscus sabdariffa* Reduces Body Fat by Inhibiting Hepatic Lipogenesis and Preadipocyte Adipogenesis,” Food & Function 7 (2016): 171–182, 10.1039/c5fo00714c. 26489044

[51] W. Liu , L. Wang , and J. Zhang , “Peanut Shell Extract and Luteolin Regulate Lipid Metabolism and Induce Browning in 3 T3‐L1 Adipocytes,” Food 11, no. 17 (2022): 2696, 10.3390/foods11172696. PMC9455591 36076880

[52] A. Sinha , N. Nosoudi , and N. Vyavahare , “Elasto‐Regenerative Properties of Polyphenols,” Biochemical and Biophysical Research Communications 444 (2014): 205–211, 10.1016/j.bbrc.2014.01.027. 24440697 PMC3947410

[53] A. Dooley , X. Shi‐Wen , N. Aden , et al., “Modulation of Collagen Type I, Fibronectin and Dermal Fibroblast Function and Activity, in Systemic Sclerosis by the Antioxidant Epigallocatechin‐3‐Gallate,” Rheumatology (Oxford, England) 49 (2010): 2024–2036, 10.1093/rheumatology/keq208. 20627968

