# Supplementary material for: Plasminogen Activator Inhibitor 1 Controls Abdominal Aortic Aneurism Formation via the Modulation of TGF‐β/Smad2/3 Signaling in Mice
Source: FASEB J. 2025 May 5;39(9):e70562. doi: 10.1096/fj.202403133RR (PMC12051826; doi:10.1096/fj.202403133RR)
Supplement: Supplementary file 1 — Data S1. [file FSB2-39-e70562-s001.pdf]

Plasminogen Activator Inhibitor 1 Controls Abdominal Aortic Aneurysm Formation via the Modulation of TGF-β/Smad2/3 signaling in Mice

***Corresponding author:**

1. Prof. Xian Wu Cheng, Department of Cardiology and Hypertension, Jilin Provincial Key Laboratory of Stress and Cardiovascular Disease, Yanbian University Hospital, 1327 Juzijie, Yanji, Jilin PR. 133000, China. Electronic address: chengxw0908@163.com

2. Prof. Lina Hu, Department of Public Health, Guilin Medical College, Guilin, Guangxi 541004, PR. China. Electronic address: 123860133@qq.com

2. Dr Meilan Liu, Department of Cardiology and Hypertension, Yanbian University Hospital, 1327 Juzijie, Yanji, Jilin PR. 133000, China. Electronic address: lan73180@163.com

Supplemental Materials

Figures S1-6

Figure legends

Figure S1. The four-week $CaCl_2$-induced AAA model decreased PAI-1 level in plasma **A**: ELISA data showing the plasma levels of PAI-1 in two groups (n=7-9/group). **B**: ELISA data showing plasma levels of TNF-α and IL-1β in two groups. (n=7/group). **C**: ELISA data showing the plasma levels of PAI-1 in three groups (n=7-9/group). **D**: Quantitative ELISA data showing plasma levels of TNF-α and IL-1β in three groups (n=7/group). Data are mean±SEM. Statistical significance was assessed by Student *t* test for A and B. Statistical significance was assessed by 1-way ANOVA for C and D.

Figure S2. PAI-1 deficiency increased mRNA levels related to inflammation, matrix protein, and proteolysis. **A-D**: representative the mRNA levels of collagen I, collagen III, MMP-2, MMP-9, $gp91^{phox}$, ICAM-1, and VCAM-1 in two groups (n=6-7/group). Data are mean±SEM. Statistical significance was assessed by Student *t* test.

Figure S3. EGCG decreased gelatinase activities and PAI-1 deficiency increased gelatinase activities. **A** and **B**: Representative zymographic images and the quantitative data of MMP-2 and MMP-9 activities in the 4 groups. (n=4/group). **C** and **D**: Representative zymographic images and the quantitative data of MMP-2 and MMP-9 activities in three groups. (n=4/group). Data are mean±SEM. Statistical significance was assessed by 1-way ANOVA for B and D.

Figure S4. PAI-1 gene was silence through siRNA transfected in VSMCs. **A**: Representative the green fluorescent was observed in MAVSMCs. Scale bar: 75μm. **B**: qPCR data show mRNA level of PAI-1 in five groups. **C** and **D**: Representative Western Blotting images and quantitative data for the levels of five groups. Data are mean±SEM. Statistical significance was assessed by 1-way ANOVA for B and D. PAI-1 siRNA 1, PAI-1 siRNA 2, and PAI-1 siRNA 3 indicate three different sequences for PAI-1 silencing. Red arrows represent positive cells with green fluorescence.

Figure S5. PAI-1 gene was overexpressed through pcDNA3.1(+) transfected in VSMCs and inflammatory factors levels in plasma. **A**: Representative the green fluorescent was observed in MAVSMCs. Scale bar: 75μm. **B**: ELISA data show the supernatant level of PAI-1 in two groups. (n=6/group) **C** and **D**: Representative Western Blotting images and quantitative data for the levels of PAI-1 in two groups. (n=3/group). Data are mean±SEM. Statistical significance was assessed by Student *t* test for B and D.

Figure S6. The proposed mechanism of how EGCG treatment prevent calcium chloride2-induced AAA formation. EGCG treatment lowered plasma TNF-α and IL-1β levels, oxidative stress production (NADPH oxidase activity), apoptosis-related protein levels (C-cas8), macrophage infiltration, and TGF-β/Smad2/3 signaling activation, which reduced vascular smooth muscle apoptosis, elastin disruption, and extracellular matrix turnover, leading to amelioration of AAA formation in mice in response to calcium chloride2-induction stress, whereas genetic and pharmacological interventions targeted toward PAI accelerated AAA formation in mice under our experimental conditions. AAA, abdominal aortic aneurysm; PAI-1, Plasminogen activator inhibitor 1; EGCG, Epigallocatechin-3-gallate; NADPH, Nicotinamide adenine dinucleotide phosphate; C-cas8, cleaved-caspase 8; TUNEL, TdT-mediated dUTP nick-end labeling; TNF-α, Tumor Necrosis Factor-α; IL-1β, Interleukin-1β; TGF-β, Transforming Growth Factor-β; MMP-2/-9, matrix metalloproteinase-2/-9. SB431542, TGFβ/Smad2/3 signaling pathway inhibitor; The red arrows represent a decreasing trend and the blue arrow represents an increasing trend.

Figure S1

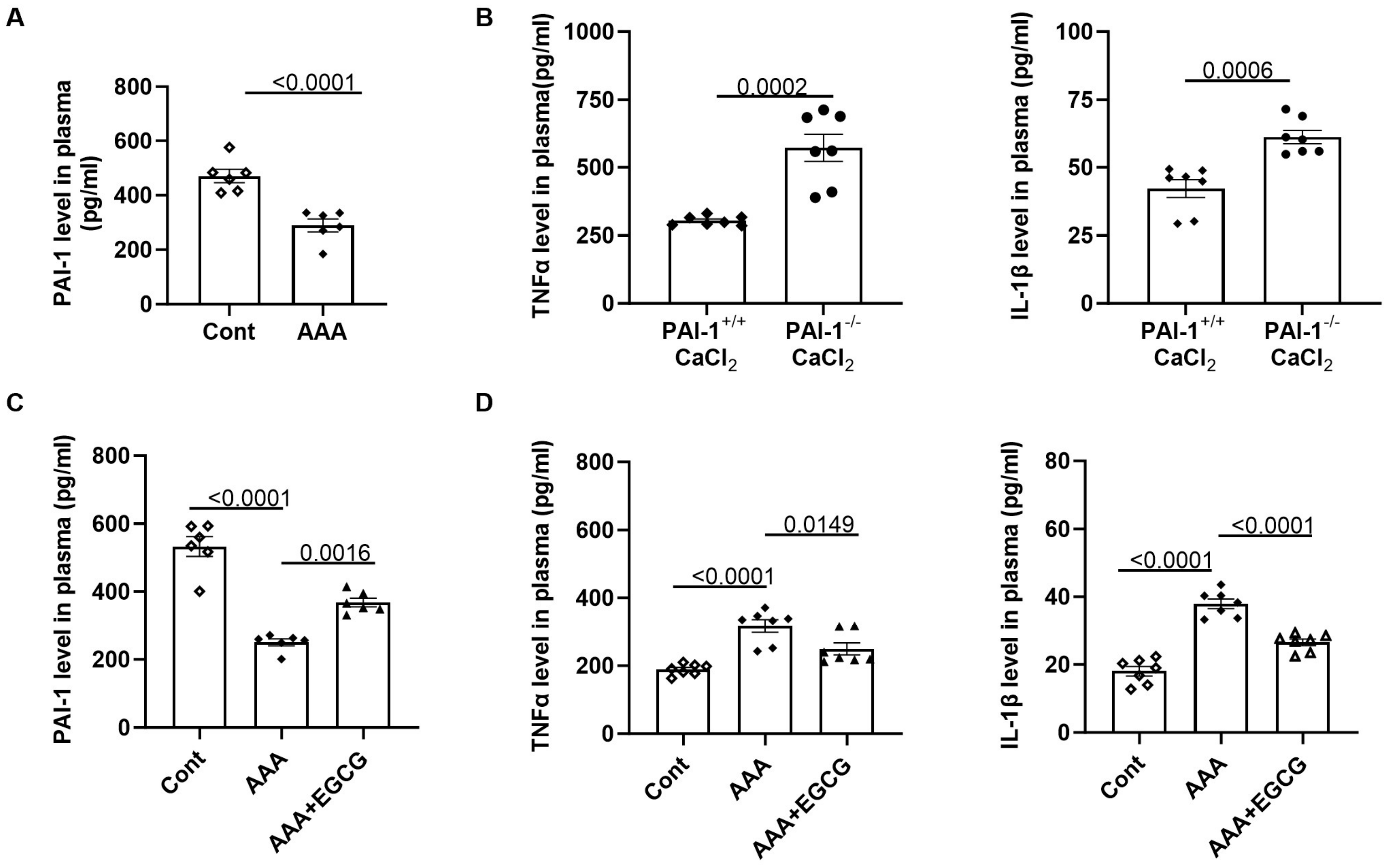
A
PAI-1 level in plasma (pg/ml)
800
600
400
200
0
<0.0001
Cont
AAA
B
TNFα level in plasma(pg/ml)
1000
750
500
250
0
0.0002
PAI-1+/+ CaCl2
PAI-1-/- CaCl2
IL-1β level in plasma (pg/ml)
100
75
50
25
0
0.0006
PAI-1+/+ CaCl2
PAI-1-/- CaCl2
C
PAI-1 level in plasma (pg/ml)
800
600
400
200
0
<0.0001
0.0016
Cont
AAA
AAA+EGCG
D
TNFα level in plasma (pg/ml)
800
600
400
200
0
<0.0001
0.0149
Cont
AAA
AAA+EGCG
IL-1β level in plasma (pg/ml)
80
60
40
20
0
<0.0001
<0.0001
Cont
AAA
AAA+EGCG

Figure S2

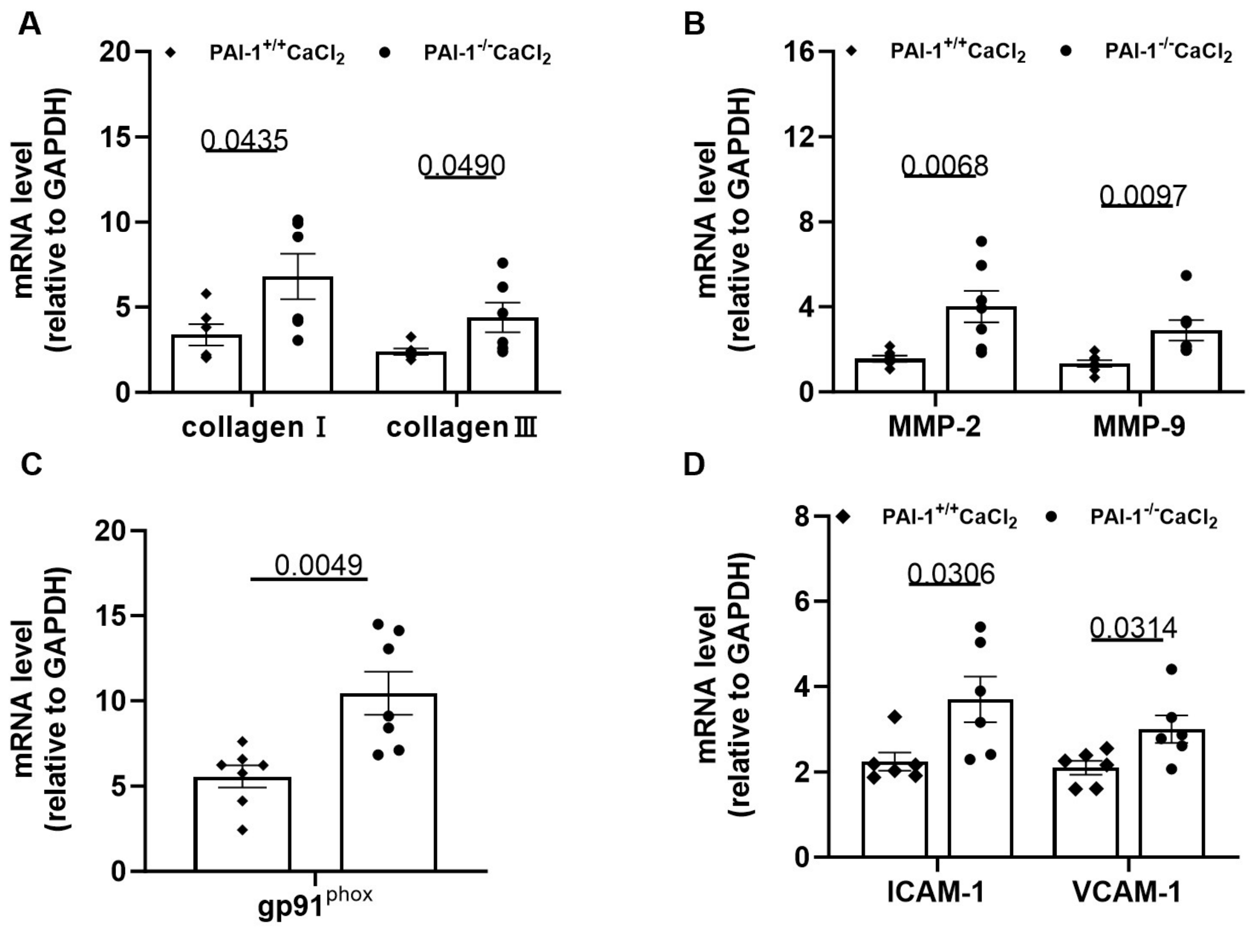
A
B
C
D
PAI-1+/+CaCl2
PAI-1-/-CaCl2
mRNA level
(relative to GAPDH)
0.0435
0.0490
collagen Ⅰ
collagen Ⅲ
0.0068
0.0097
MMP-2
MMP-9
0.0049
gp91phox
0.0306
0.0314
ICAM-1
VCAM-1

Figure S3

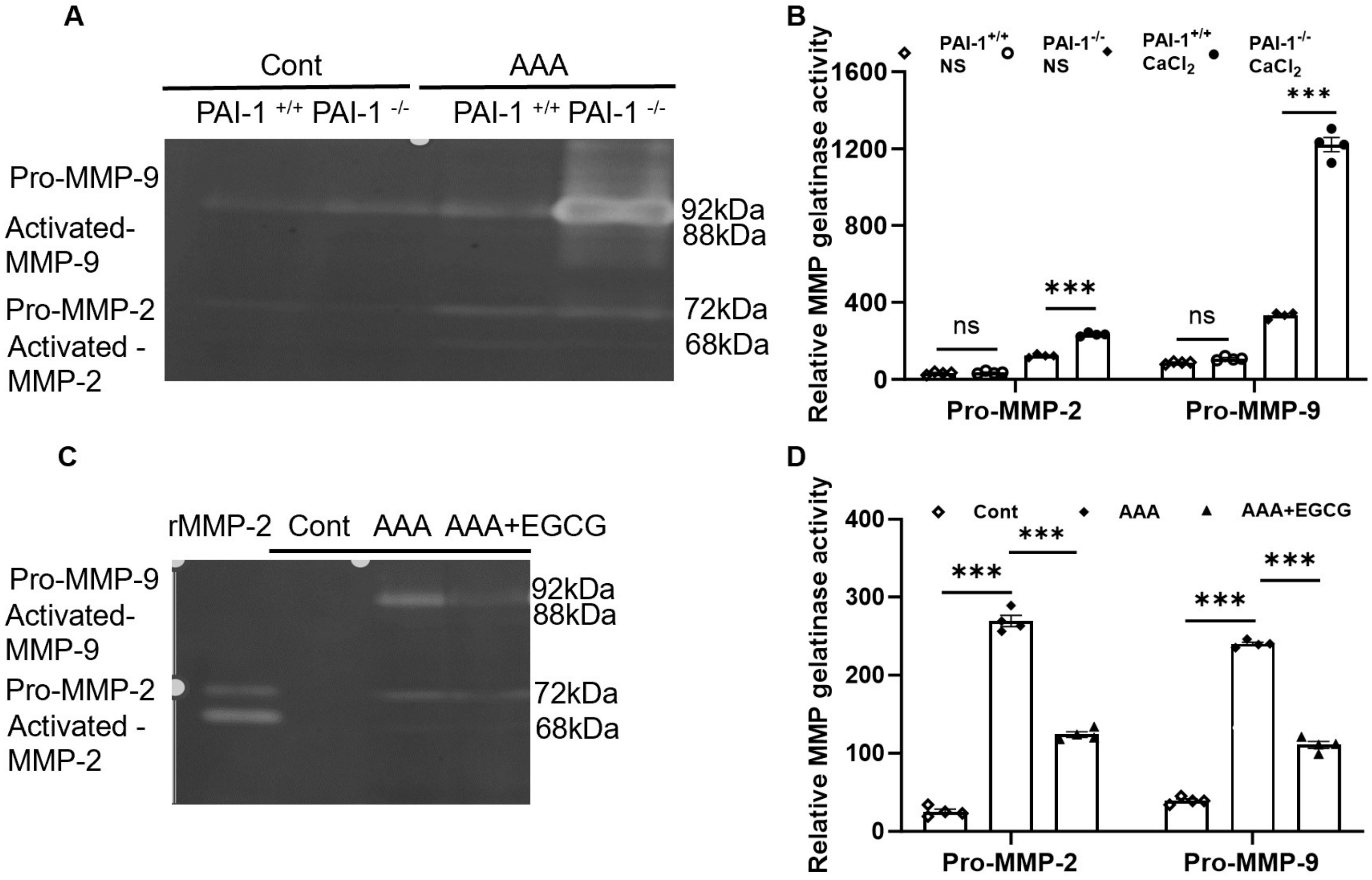
A
Cont
AAA
PAI-1 +/+ PAI-1 -/-
PAI-1 +/+ PAI-1 -/-
Pro-MMP-9
Activated-MMP-9
Pro-MMP-2
Activated -MMP-2
92kDa
88kDa
72kDa
68kDa
B
PAI-1+/+ NS
PAI-1-/- NS
PAI-1+/+ CaCl2
PAI-1-/- CaCl2
Relative MMP gelatinase activity
1600
1200
800
400
0
ns
***
ns
***
Pro-MMP-2
Pro-MMP-9
C
rMMP-2
Cont
AAA
AAA+EGCG
Pro-MMP-9
Activated-MMP-9
Pro-MMP-2
Activated -MMP-2
92kDa
88kDa
72kDa
68kDa
D
Cont
AAA
AAA+EGCG
Relative MMP gelatinase activity
400
300
200
100
0
***
***
***
***
Pro-MMP-2
Pro-MMP-9

Figure S4

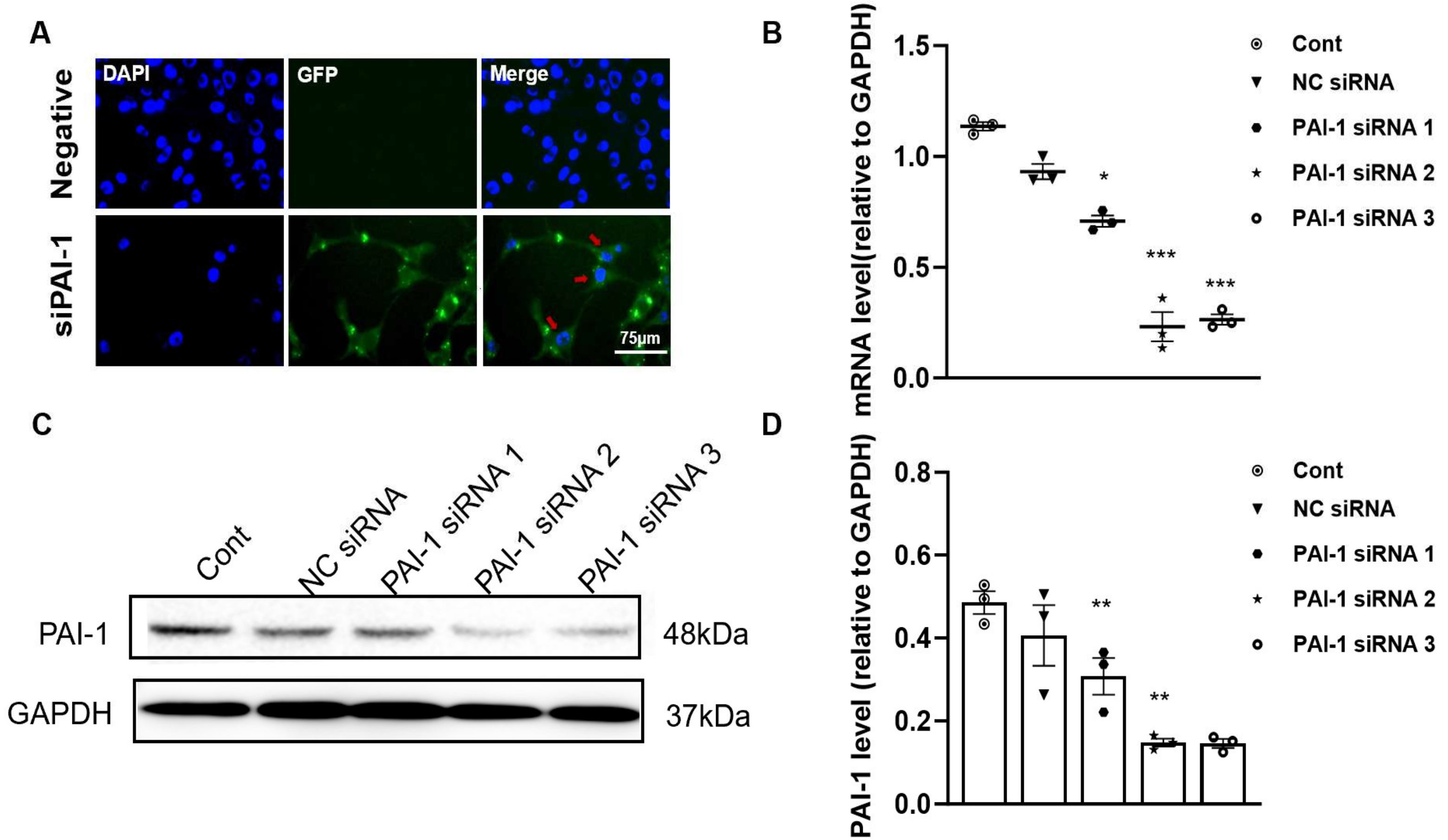
A
DAPI
GFP
Merge
Negative
siPAI-1
75μm
B
mRNA level(relative to GAPDH)
1.5
1.0
0.5
0.0
Cont
NC siRNA
PAI-1 siRNA 1
PAI-1 siRNA 2
PAI-1 siRNA 3
*
***
***
C
Cont
NC siRNA
PAI-1 siRNA 1
PAI-1 siRNA 2
PAI-1 siRNA 3
PAI-1
48kDa
GAPDH
37kDa
D
PAI-1 level (relative to GAPDH)
0.8
0.6
0.4
0.2
0.0
Cont
NC siRNA
PAI-1 siRNA 1
PAI-1 siRNA 2
PAI-1 siRNA 3
**
**

Figure S5

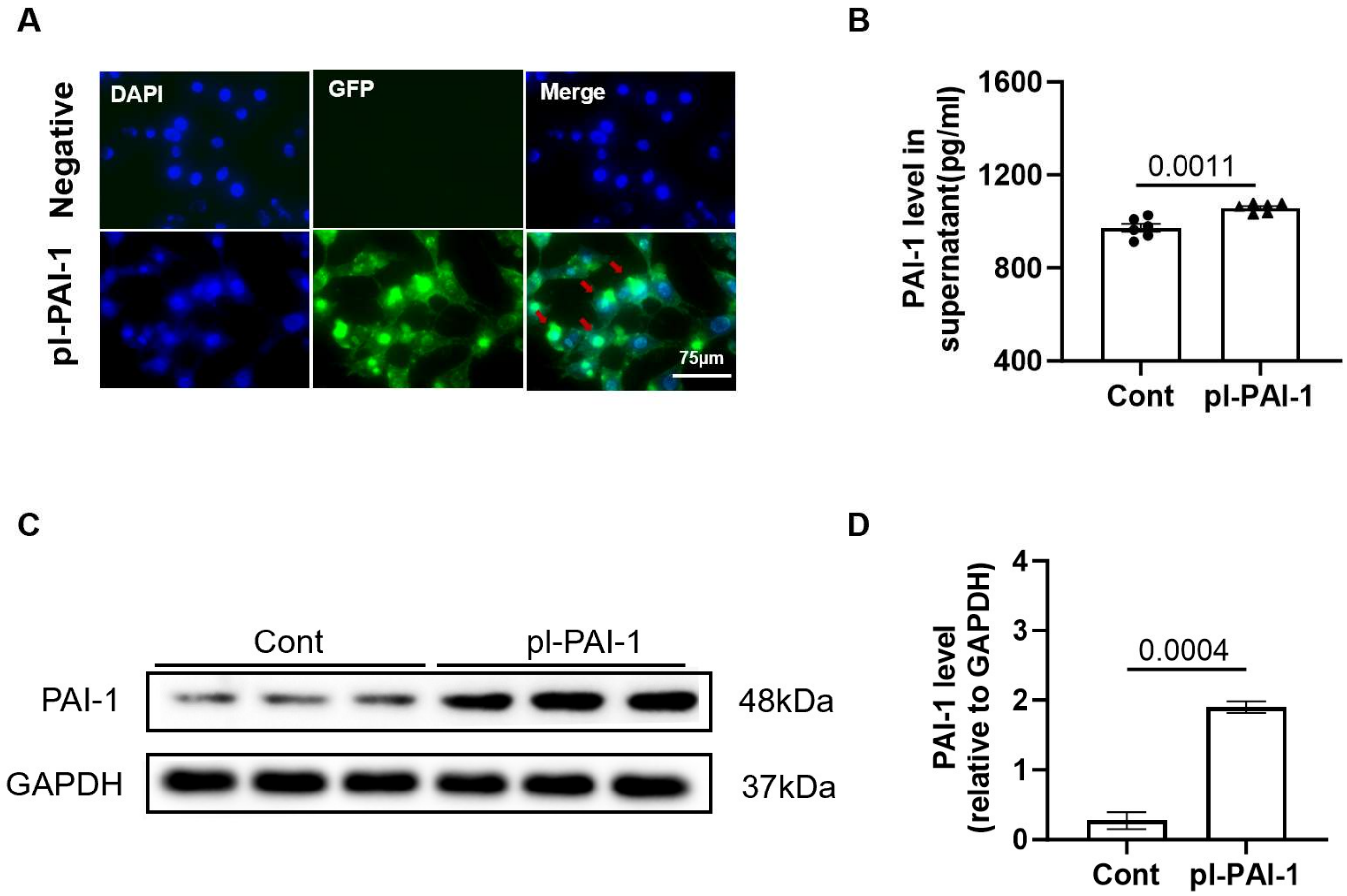
A
DAPI
GFP
Merge
Negative
pl-PAI-1
75μm
B
PAI-1 level in supernatant(pg/ml)
1600
1200
800
400
0.0011
Cont
pl-PAI-1
C
Cont
pl-PAI-1
PAI-1
48kDa
GAPDH
37kDa
D
PAI-1 level (relative to GAPDH)
4
3
2
1
0
0.0004
Cont
pl-PAI-1

Figure S6

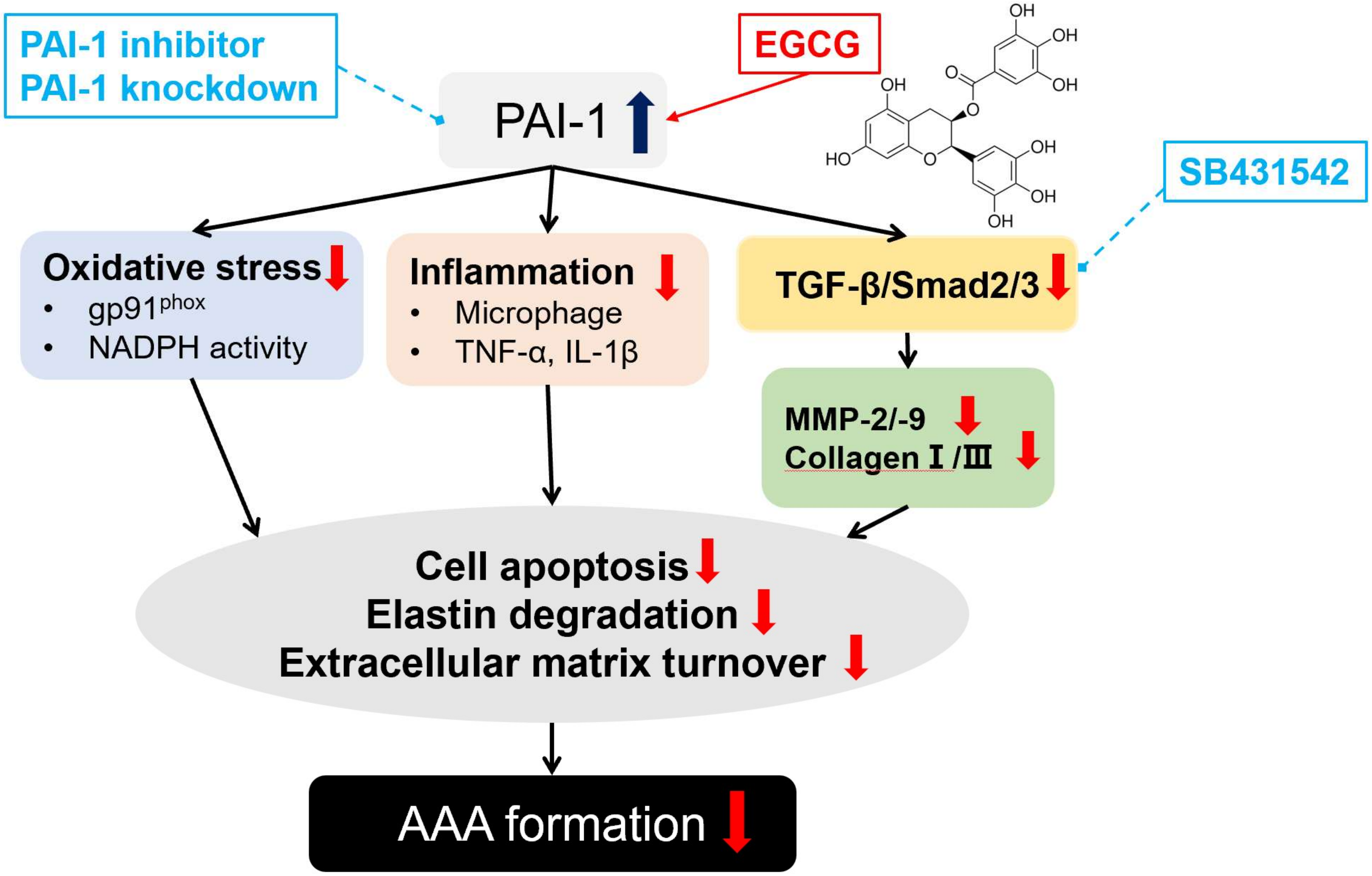
PAI-1 inhibitor
PAI-1 knockdown
EGCG
PAI-1
SB431542
OH
OH
O
OH
OH
O
HO
O
OH
OH
OH
Oxidative stress
• gp91phox
• NADPH activity
Inflammation
• Microphage
• TNF-α, IL-1β
TGF-β/Smad2/3
MMP-2/-9
Collagen Ⅰ/Ⅲ
Cell apoptosis
Elastin degradation
Extracellular matrix turnover
AAA formation
